# Tumor‐Informed ctDNA in Guiding First‐Line Immunochemotherapy in Advanced Non‐Small Cell Lung Cancers

**DOI:** 10.1002/advs.202506565

**Published:** 2025-11-25

**Authors:** Kailun Fei, Jie Zhao, Jiachen Xu, Jianchun Duan, Chengcheng Li, Wenchuan Xie, Jia Zhong, Guoqiang Wang, Yu Xu, Boyang Sun, Rui Wan, Hua Bai, Qinxiang Guo, Wei Guo, Shangli Cai, Zhijie Wang, Jie Wang

**Affiliations:** ^1^ State Key Laboratory of Molecular Oncology CAMS Key Laboratory of Translational Research on Lung Cancer Department of Medical Oncology National Cancer Center/National Clinical Research Center for Cancer/Cancer Hospital Chinese Academy of Medical Sciences and Peking Union Medical College Beijing P. R. China; ^2^ Burning Rock Biotech Guangdong P. R. China; ^3^ Department of Respiratory Medicine Shanxi Province Cancer Hospital/ Shanxi Hospital Affiliated to Cancer Hospital Chinese Academy of Medical Sciences/Cancer Hospital Affiliated to Shanxi Medical University ShanXi P. R. China

**Keywords:** chemotherapy, immune checkpoint inhibitors, predictive biomarker, tumor‐informed ctDNA

## Abstract

Predictive biomarkers are urgently needed for first‐line immune checkpoint inhibitors plus chemotherapy (ICI‐chemo) in advanced non‐small cell lung cancer (NSCLC). Circulating tumor DNA (ctDNA) reflects tumor burden and immunogenicity, potentially identifyinging of patients suitable for ICI‐chemo. In this study, pre‐ and on ‐ treatment tissue and plasma samples are prospectively collected and analyzed from the randomized phase III CHOICE‐01 trial comparing ICI ‐ chemo versus chemotherapy alone in advanced NSCLC. Pre‐treatment tissue and plasma samples, as well as on‐treatment plasma samples, are prospectively collected. Tumor‐informed ctDNA detection is based on tissue‐identified mutations. Among patients with tumor‐informed ctDNA positivity, those receiving ICI‐chemo experienced significantly improved‐free survival (PFS) and overall survival (OS) compared to those receiving chemotherapy alone (PFS: HR 0.45, 95% CI 0.34–0.60; OS: HR 0.66, 95% CI 0.49–0.88; p = 0.0045). In contrast, no significant differences in PFS or OS are observed between treatment arms in the ctDNA‐negative subgroup. The predictive value of tumor‐informed ctDNA is independent of other immune biomarkers and superior to other ctDNA metrics. Validation in a combined cohort from RATIONALE 304/307 trials shows similar results. Furthermore, ctDNA clearance during treatment correlates with better clinical outcomes (log‐rank p = 0.0004 for OS and p = 0.044 for PFS).

## Graphical abstract

Tumor‐informed circulating tumor DNA (ctDNA) analysis identifies patients with advanced non‐small cell lung cancer (NSCLC) who benefit from first‐line immunotherapy plus chemotherapy. Longitudinal ctDNA monitoring further reflects therapeutic response and predicts prognosis beyond imaging. These findings support ctDNA as a practical and dynamic biomarker to guide treatment selection and real‐time assessment of clinical benefit in advanced NSCLC.

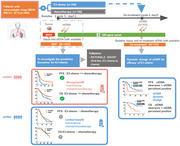

## 1 Introduction

Immune checkpoint inhibitors (ICIs) combined with chemotherapy (ICI‐chemo) have been approved as the first‐line treatment for advanced non‐small cell lung cancer (NSCLC), demonstrating an improvement in progression‐free survival (PFS) and overall survival (OS).^[^ ^1^, ^2^, ^3^, ^4^ ^]^ However, not all patients respond equally to ICI‐chemo, which underscores the urgent need for predictive biomarkers to identify patients who are most likely to benefit from this treatment.

Significant efforts have been made to explore the predictive biomarkers for ICI‐chemo. However, the biomarkers that were previously well‐established in ICI monotherapy have proven ineffective in informing treatment benefit from ICI‐chemo.^[^ ^1^, ^2^, ^3^, ^4^, ^5^ ^]^ For instance, tissue tumor mutation burden (TMB) and programmed death‐ligand 1 (PD‐L1) expression have shown limited efficacy in predicting a superior survival benefit from ICI‐chemo over chemotherapy alone in the first‐line setting.^[^ ^1^, ^2^, ^3^, ^4^, ^5^ ^]^ Moreover, most studies investigating biomarkers for ICIs did not include a control group. Consequently, it remains uncertain whether the identified biomarkers are purely prognostic or truly predictive and can guide treatment selection.

In recent years, circulating tumor DNA (ctDNA) testing has emerged as a potential biomarker to transform patient management by providing real‐time assessments of patient prognoses and responses to treatment.^[^ ^6^, ^7^, ^8^ ^]^ In early‐stage cancer, detectable ctDNA is likely indicative of molecular residual disease (MRD), necessitating more intensive treatment. In advanced‐stage cancer, patients with ctDNA clearance, defined as undetectable ctDNA during treatment, have a longer survival after receiving ICIs or ICI‐chemo treatment.^[^ ^9^, ^10^, ^11^ ^]^ Moreover, ctDNA release is strongly associated with tumor immunogenicity.^[^ ^12^, ^13^ ^]^ For example, ctDNA shedding has been linked to upregulated cell cycle signaling, chromosomal instability, decreased DNA damage repair, and elevated neoantigen levels,^[^ ^12^, ^13^ ^]^ which may be associated with enhanced antitumor immune responses. Several studies have also suggested that ctDNA may serve as a predictive biomarker for ICIs.^[^ ^13^, ^14^ ^]^ In locally advanced NSCLC, consolidation immunotherapy yields significantly better survival than placebo in patients with positive ctDNA rather than in those with negative ctDNA.^[^ ^14^ ^]^ Similarly, in urothelial carcinoma, adjuvant immunotherapy is associated with improved outcomes compared with observation only in patients who have positive ctDNA post‐surgery.^[^ ^13^ ^]^ Furthermore, the peripheral ctDNA burden may be sensitive to chemotherapy‐induced immunogenic cell death (ICD), thereby providing ICI with additional benefits in the combination regimen.^[^ ^15^ ^]^ Altogether, these results suggest that ctDNA may be a potential biomarker to indicate benefit from ICI‐chemo in advanced NSCLC.

The technologies applied for ctDNA detection can be classified as tumor‐informed and tumor naïve approaches.^[^ ^16^, ^17^, ^18^, ^19^ ^]^ The tumor‐informed approach involves sequencing tumor tissue to identify patient‐specific mutations that are subsequently tracked in plasma. In contrast, the tumor‐naïve approach does not require prior tissue‐sequencing information and directly tests the plasma. In advanced‐stage NSCLC, the sensitivity and specificity of plasma ctDNA detection using the tumor‐naïve approach are impeded by non‐shedding tumors and apoptotic hematopoietic cells with stochastic somatic alterations, a phenomenon known as clonal hematopoiesis of indeterminate potential (CHIP), respectively.^[^ ^20^, ^21^, ^22^ ^]^ Conversely, the tumor‐informed approach, which leverages tumor biopsy samples to identify patient‐specific mutations, exhibits a lower limit of detection (LOD).^[^ ^20^, ^23^ ^]^ and is less affected by CHIP.^[^ ^20^, ^24^ ^]^ Thus, in the present study, we aimed to study whether the tumor‐informed ctDNA was associated with the benefit from ICI‐chemo treatment.

The CHOICE‐01 study was a randomized, placebo‐controlled trial assessing the efficacy and safety of the programmed death‐1 (PD‐1) antibody toripalimab in combination with chemotherapy in treatment‐naive patients with stage IIIB‐IV NSCLC, and met its primary endpoint.^[^ ^3^ ^]^ In this study, we investigated the predictive role of tumor‐informed ctDNA status in identifying patients who derive superior survival benefit from ICI‐chemo compared to chemotherapy alone. The predictive role of tumor‐informed ctDNA status for ICI‐chemo was further validated in another cohort merged by two randomized phase III trials, the RATIONALE 304 and 307 studies.^[^ ^25^, ^26^ ^]^ Additionally, on‐treatment plasma samples were used to explore whether dynamic changes of ctDNA could predict therapeutic efficacy of ICI‐chemo.

## 2 Results

### 2.1 Study Design and Patient Characteristics

**Figure 1:**
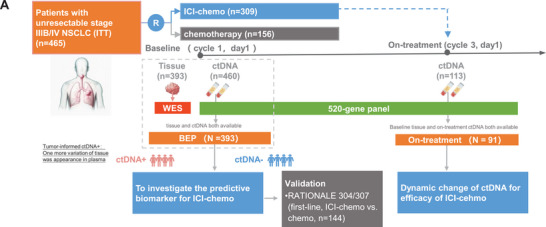
Study schema and the comparison of ITT and BEP. Schema of analysis. Specifically, a total of 465 patients were enrolled in the CHOICE‐01 study and randomized at a 2:1 ratio to the PD1 inhibitor combined with chemotherapy and the chemotherapy arm. Among them, 393 patients with tumor samples and plasma samples were defined as the biomarker evaluable population. Blood samples were collected at Day 1 at 1st cycling (C1D1) and at Day 1 at 3rd cycling (C3D1). The tissue samples were sequenced by whole‐exon sequencing, and blood samples were sequenced with a 520‐gene panel‐based deep sequencing.

The study design is illustrated in **Figure** **1**A. The primary objective was to investigate the predictive role of tumor‐informed ctDNA for ICI‐chemo in the CHOICE‐01 study. Another two randomized phase III trials in advanced NSCLC, the RATIONALE 304 (NCT03663205) and RATIONALE 307 (NCT03594747), were merged as a validation cohort.^[^ ^25^, ^26^ ^]^

In the CHOICE‐01 study,^[^ ^3^ ^]^ a total of 465 patients were enrolled and randomized, defined as the intention‐to‐treat (ITT) population. Tissue and plasma samples were collected prospectively before and during study‐indicated treatment. A subset of patients (393/465, 84.5%) who had both baseline tissue and plasma samples successfully sequenced was defined as the biomarker evaluation population (BEP). The baseline characteristics, including Eastern Cooperative Oncology Group (ECOG) performance status, age, sex, and histology, were comparable between the BEP and ITT populations (Table S1, Supporting Information). The present analysis was based on the final updated OS (data cutoff August 31, 2022).^[^ ^27^ ^]^ Survival outcomes were similar between the ITT and BEP populations (OS, HR = 1.05 [0.88–1.25]; PFS, HR = 1.02 [0.87–1.19]; Figure S1A,B, Supporting Information). Moreover, no survival benefit bias was observed when comparing ICI‐chemo versus chemotherapy in both ITT and BEP (Figure S1C–F, Supporting Information).

### 2.2 Comparison of Tumor‐Informed ctDNA with Tumor‐Naïve ctDNA

Previous studies have demonstrated that tumor‐informed methods exhibit a lower limit of detection (LOD) and are less influenced by clonal hematopoiesis (CH) than tumor‐naïve methods.^[^ ^17^, ^20^, ^21^, ^22^ ^]^ In our study, we first compared these approaches using pretreatment plasma samples from the CHOICE‐01 study (**Methods**).

Among the BEP samples, 87.7% (345 out of 393) exhibited concordant ctDNA status by the two approaches (Figure S2A, Supporting Information). However, 13 samples were positive exclusively via the tumor‐informed approach (tumor‐informed ctDNA+/tumor‐naïve ctDNA‐), and 35 samples were positive only by the tumor‐naïve approach (tumor‐informed ctDNA‐/tumor‐naïve ctDNA+) (Figure S2A, Supporting Information). The maximum somatic allele fraction (MSAF) for the 13 samples with tumor‐informed ctDNA+/tumor‐naïve ctDNA‐ was as low as 0.07%, substantially lower than 0.5%, the LOD of the tumor‐naïve approach (Figure S2B, Supporting Information). In the absence of paired tissue samples, these samples with low‐level ctDNA were erroneously classified as negative using the tumor‐naïve approach, suggesting the enhanced sensitivity of the tumor‐informed method.

On the other hand, among the 94 patients who were tumor‐informed ctDNA‐negative, 35 (37.2%) were positive by tumor‐naïve ctDNA analysis. In this subset, the median allele fraction (AF) was 0.93% (Figure S2C, Supporting Information). Besides, among all the genes mutated (n = 88) in the plasma samples of the 35 patients, 51 genes (58.0%) were mutated only in plasma but not in paired tissue samples or other tissue samples in the BEP (Figure S2D,E and Table S2, Supporting Information). Of these 51 genes, 21 were linked to CH (Table S3, Supporting Information), including *NOTCH1, DNMT3A*, *IDH2, CALR*, and *CCND3*. Notably, 37.1% (13 out of 35) of the patients only harbored CH‐associated mutations in their plasma (Table S2, Supporting Information), suggesting that several positive results identified by the tumor‐naïve approach may be potential false positives due to CH. These results indicate that the tumor‐informed approach reduces the risk of false negatives and false positives.

### 2.3 Tumor‐Informed ctDNA Predicts a Superior Survival Benefit of ICI‐Chemo Over Chemotherapy

We further compared the predictive value of tumor‐informed and tumor‐naïve ctDNA for ICI‐chemo efficacy. Among the BEP patients, 321 out of 393 (81.7%) exhibited detectable ctDNA in the plasma with the tumor‐naïve approach at baseline (C1D1). Tumor‐naïve ctDNA+ was associated with shorter PFS and OS (Figure S3A,B, Supporting Information). However, it was not associated with improved outcomes for patients treated with ICI‐chemo compared to those treated with chemotherapy alone (PFS and OS interaction p > 0.05; Figure S3A,B, Supporting Information). MSAF and ctDNA concentration (mean tumor molecules per ml, MTM/ml) across various cutoffs (10th to 90th percentile) similarly failed to show a predictive value for prolonged survival with ICI‐chemo (interaction p > 0.05; Figure S3C,D and Table S4, Supporting Information). These results suggest that ctDNA metrics using the tumor‐naïve approach are prognostic rather than predictive, which is consistent with the previous studies.^[^ ^13^ ^]^

**Figure 2:**
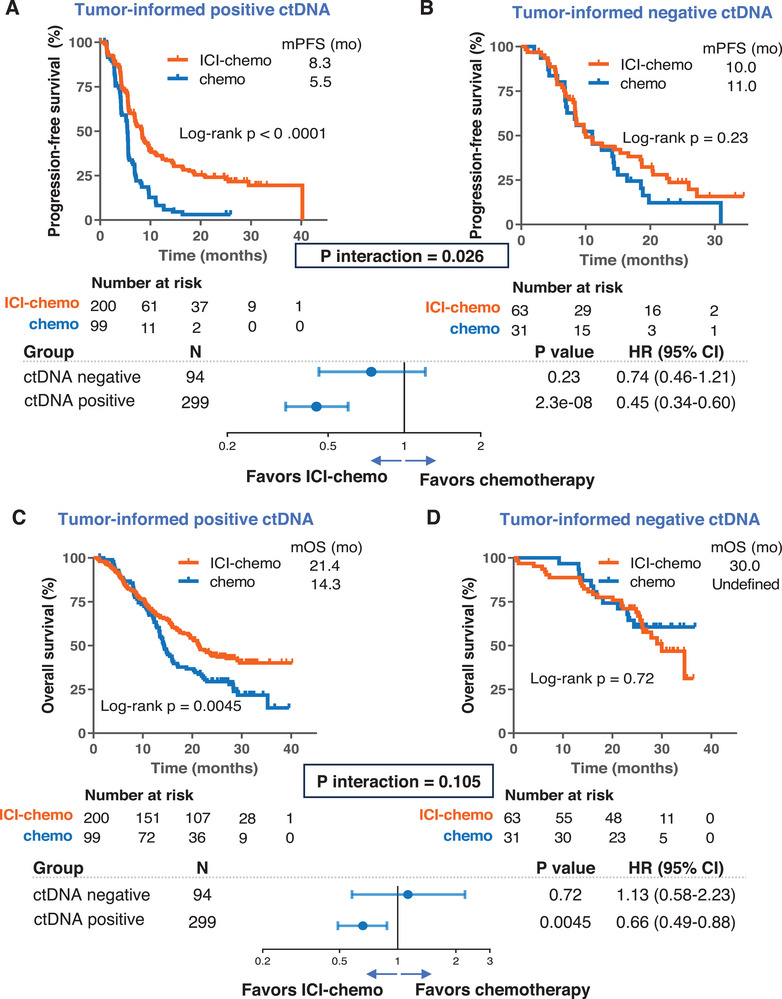
Predictive role of tumor‐informed ctDNA in the CHOICE‐01 cohort. A,B). Kaplan‐Meier curves comparing progression‐free survival (PFS) between ICI‐chemo and chemotherapy in patients with ctDNA‐negative or ctDNA‐positive status. Upper, Kaplan‐Meier curves depicting the treatment comparison; lower, forest plot depicting the relative efficacy of ICI‐chemo versus chemotherapy. C,D). Kaplan‐Meier curves comparing overall survival (OS), ICI‐chemo, and chemotherapy in patients with tumor‐informed ctDNA‐ or tumor‐informed ctDNA+. Upper, Kaplan‐Meier curves depicting the treatment comparison; lower, forest plot depicting the relative efficacy of chemo‐ICI versus chemotherapy alone. The hazard ratios, corresponding 95% confidence intervals, and statistical significance of the difference were computed using the Cox proportional hazards model.

In contrast, tumor‐informed ctDNA was positive in 76.1% (299 out of 393) of patients at baseline. In patients with positive tumor‐informed ctDNA (tumor‐informed ctDNA+), ICI‐chemo was associated with significantly prolonged PFS compared to chemotherapy (HR = 0.45 [95% CI: 0.34–0.60]; p < 0.001; **Figure** **2**A). Conversely, in patients with negative tumor‐informed ctDNA (tumor‐informed ctDNA‐), no statistically significant difference in PFS was observed between the ICI‐chemo and chemotherapy groups (PFS, HR = 0.74 [95% CI: 0.46–1.21]; p = 0.23; Figure 2B). Notably, similar trends were observed for OS in that the ICI‐chemo regimen conferred superior outcomes compared with chemotherapy in the tumor‐informed ctDNA+ subgroup (HR = 0.66 [95% CI: 0.49–0.88]; p = 0.0045; Figure 2C), whereas no significant difference in OS between the ICI‐chemo and chemotherapy groups was detected in the tumor‐informed ctDNA‐ subgroup (HR = 1.13 [95% CI: 0.58–2.23]; p = 0.72; Figure 2D). The interaction between therapeutic regimens (ICI‐chemo vs chemotherapy) and tumor‐informed ctDNA status showed significance for PFS, but the difference did not reach statistical significance for OS (p interaction = 0.026 for PFS, p interaction = 0.105 for OS, Figure 2A–D), suggesting a specific predictive role of tumor‐informed ctDNA status for superior PFS of ICI‐chemo over chemotherapy alone. In multivariable Cox regression by adjusting for PD‐L1 expression, histology, smoking status, and age, the predictive role of tumor‐informed ctDNA remained significant for PFS (p interaction = 0.032), Table S5 (Supporting Information).

We further studied the associated factors of ctDNA release. Smokers, prior adjuvant treatment, larger tumor sizes, and lung squamous carcinoma were positively associated with tumor‐informed ctDNA+ (p < 0.05; Table S6). In contrast, tumor‐informed ctDNA was associated with adenocarcinoma, non‐smokers, absence of prior adjuvant treatment, and smaller tumor sizes (p < 0.05; Table S6). Detailed clinical‐pathological characteristics of this subgroup are provided in Table 7 (Supporting Information).

In subgroup analysis stratified by clinical variables, including ECOG performance status, PD‐L1 expression, histology, smoking status, and visceral or bone metastasis status, the treatment effect on PFS and OS favored the ICI‐chemo arm over the chemotherapy arm across most subgroups in the tumor‐informed ctDNA+ group (Figure S4A,B, Supporting Information). In contrast, no significant difference in the treatment effect was observed between ICI‐chemo and chemotherapy in all subgroups of the tumor‐informed ctDNA group (Figure S4A,B, Supporting Information). These results suggest that tumor‐informed ctDNA status can predict a superior survival benefit for ICI‐chemo over chemotherapy across most clinical subgroups.

We further compared the predictive value of tumor‐informed and tumor‐naïve approaches for ICI‐chemo efficacy in samples with inconsistent results. Among patients who were tumor‐informed ctDNA‐ but tumor‐naïve ctDNA+ (N = 35), there were no significant differences in OS between the ICI‐chemo and chemotherapy groups (log‐rank p = 0.87; Figure S3E, Supporting Information), which was consistent with patients who were ctDNA‐ by both methods (log‐rank p = 0.61; Figure S3F, Supporting Information). In contrast, among patients who were tumor‐informed ctDNA+ but tumor‐naïve ctDNA‐(N = 13), OS tended to be longer for those receiving ICI‐chemo (HR = 0.34, 95% CI: 0.07‐1.72, log‐rank p = 0.192; Figure S3G, Supporting Information), which was consistent with patients who were ctDNA+ in both methods (log‐rank p = 0.003; Figure S3H, Supporting Information). Even in patients with low ctDNA concentrations (MSAF < 1%), positive tumor‐informed ctDNA predicted better survival benefits from ICI‐chemo over chemotherapy (PFS and OS, interaction p < 0.05; Figure S3I, Supporting Information). These results suggest that the predictive role of ctDNA status detected by the tumor‐informed approach for ICI‐chemo surpasses tumor‐naïve approaches.

### 2.4 Association Between Tumor‐Informed ctDNA and Chromosomal Instability and Immunogenicity

**Figure 3:**
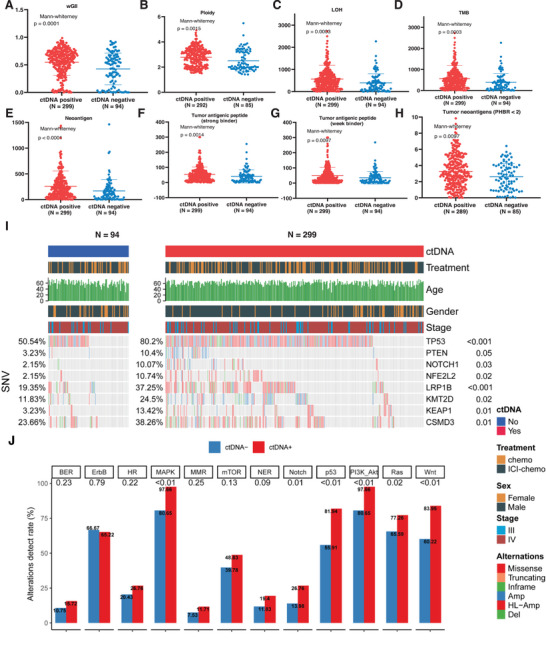
Genomic heterogeneity contributing to the tumor‐informed ctDNA status at baseline. A–H). Comparison the weighted genome instability index A), ploidy B), loss of heterozygosity C), tumor mutation burden (D), neoantigen (E), tumor antigenic peptide‐strong binder (F), tumor antigenic peptide‐weak binder (G), and high‐affinity tumor neoantigens (H) between the tumor‐informed ctDNA+ and ctDNA‐ groups. P values were calculated by the Mann‐Whitney U test. I. Oncoprint showing the alterations of driver mutation in the tumor‐informed ctDNA+ and tumor‐informed ctDNA‐ groups. P values were calculated by the Chi‐squared test. J). Comparison of altered mutation pathways between the tumor‐informed ctDNA+ and ctDNA‐ groups. P values were calculated by the Chi‐squared test.

To unveil the genomic factors influencing ctDNA status, we first compared the differences in genomic instability characteristics between the tumor‐informed ctDNA+ and ctDNA‐ groups. As a result, the weighted genomic instability index (wGII), ploidy, and loss of heterozygosity (LOH) were significantly higher in the positive ctDNA group than in the negative ctDNA group (*Mann‐Whitney*, p < 0.05; **Figure** **3**A–C). We further investigated the immunogenicity between the tumor‐informed ctDNA+ and ctDNA‐ groups. Consequently, TMB and neoantigen levels were significantly higher in the ctDNA+ group than in the ctDNA‐ group (*Mann‐Whitney*, p < 0.05; Figure 3D,E). Moreover, the amounts of predicted tumor antigenic peptides (strong or weak‐binding peptides with MHC class I) and predicted antigenic oncogenic mutations were significantly elevated in the tumor‐informed ctDNA+ group (*Mann‐Whitney*, p < 0.05; Figure 3F–H). These results suggest that the tumor‐informed ctDNA+ was associated with increased chromosomal instability and immunogenicity, which is consistent with previous studies.^[^ ^12^ ^]^

We further analyzed single‐nucleotide variations (SNVs) between the tumor‐informed ctDNA+ and ctDNA‐ groups. Mutations in a few driver genes, including *TP53*, *KEAP1, NOTCH1*, and *LRP1B*, exhibited higher mutation frequencies in the tumor‐informed ctDNA+ group than in the tumor‐informed ctDNA‐ group (*Chi‐Square test*, p < 0.05; Figure 3I). *TP53* was the most frequently mutated gene after FDR adjustment (p < 0.001; Table S8, Supporting Information). Mutations in MAPK, Notch, p53, and Wnt signaling were also significantly altered in the tumor‐informed ctDNA+ group (*Chi‐Square test*, p < 0.05; Figure 3J). However, we also observed several druggable pathway alterations in the tumor‐informed ctDNA group, such as ERBB2, MMR, and mTOR signaling (Figure 3J), suggesting that alternative strategies beyond immunotherapy might be considered.

### 2.5 The Predictive Role of Tumor‐Informed ctDNA for ICI‐Chemo is Independent of Established Immune Biomarkers

Given that tumor‐informed ctDNA+ status was associated with increased immunogenicity, we further investigated the potential confounding role of other immune biomarkers. In the tumor‐informed positive ctDNA population, tumor purity and baseline characteristics, including immune biomarkers such as PD‐L1 protein expression, TMB levels, wGII, neoantigen, ploidy, and LOH, were balanced between the ICI‐chemo arm and chemotherapy arms (Figure S5A–G, Supporting Information).

**Figure 4:**
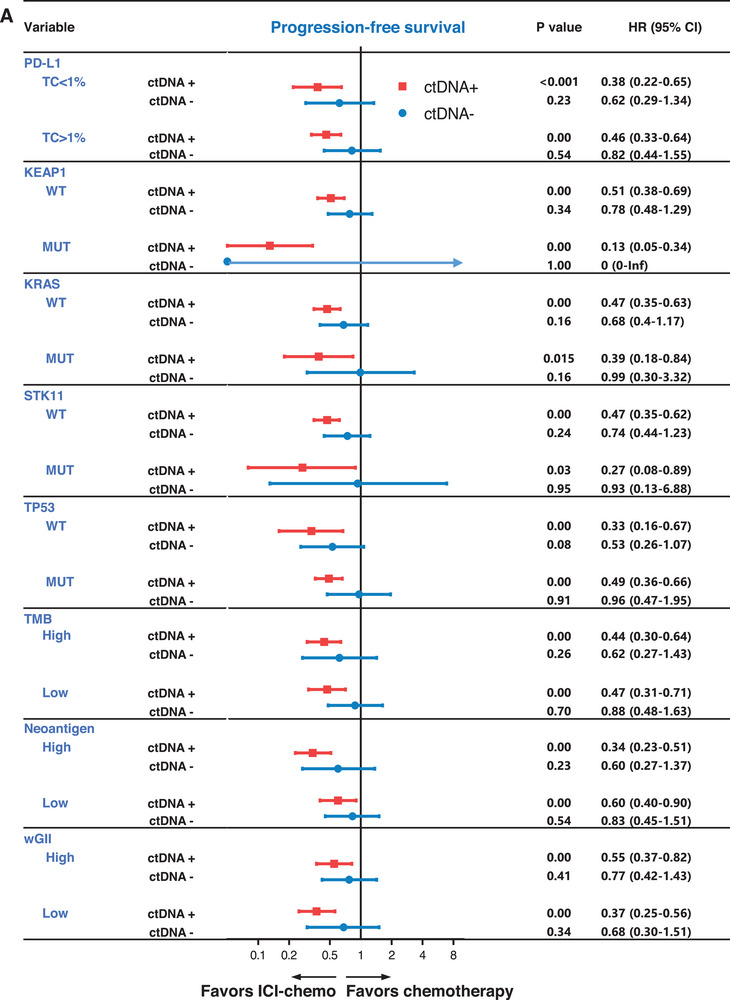
Predictive role of tumor‐informed ctDNA in the context of immune biomarkers. Comparison of PFS between ICI‐chemo and chemotherapy in tumor‐informed ctDNA+ and tumor‐informed ctDNA‐ groups, respectively, according to subgroups by immune biomarkers. P values were calculated using the log‐rank test. The HRs, HR interaction, corresponding 95% CIs, and statistical significance of the difference between the groups were computed using the Cox proportional hazards model.

To ascertain whether the predictive role of tumor‐informed ctDNA status in distinguishing the superiority of ICI‐chemo over chemotherapy was confounded by other immune biomarkers, we conducted a comprehensive investigation of therapeutic outcomes between ICI‐chemo and chemotherapy in subgroup analyses stratified by various immune biomarkers. Irrespective of subgroup classification (high or low PD‐L1, TMB, or neoantigen levels), patients in the tumor‐informed ctDNA+ group exhibited superior PFS and OS with ICI‐chemo compared to chemotherapy (log‐rank p < 0.05). However, these results were not observed in the tumor‐informed ctDNA group (**Figure** **4**A; Figure S6A, Supporting Information). A similar pattern of superior outcomes in the ctDNA‐positive group was noted across both high and low wGII groups, and in groups characterized by driver mutation status (KEAP1, STK11, KRAS, TP53; wild‐type and mutant) (Figure 4A; Figure S6A, Supporting Information).

Collectively, these findings underscore the predictive superiority of tumor‐informed ctDNA status for ICI‐chemo over chemotherapy, independent of other immune biomarkers, and highlight the potential role of tumor‐informed ctDNA as an independent predictive biomarker in this therapeutic context.

### 2.6 Potential Confounding Factors for Tumor‐Informed ctDNA Detection

In this study, the majority of tissue samples were obtained from post‐metastatic biopsies (83.0%, 326 of 393), while a smaller proportion (17.0%, 67 of 393) were derived from prior surgical specimens. The tumor‐informed ctDNA positivity rates were 77.6% and 68.7% for post‐metastatic biopsy and surgical samples, respectively (Fisher's exact test p = 0.50), suggesting that the type of tissue sample does not significantly affect the detection rate of ctDNA. The tumor‐informed ctDNA approach minimizes false positivity by identifying patient‐specific variants.^[^ ^12^ ^]^ however, false negatives may arise from two aspects: heterogeneity of advanced‐stage tumors and ultra‐low AF undetectability due to technical limitations. We further evaluated the influence of potential false negatives because of these two aspects.

On one hand, in the tumor‐informed ctDNA‐ group, 90.4% of patients (85/94) had MSAF of less than 2.0%, and only 5 patients (5/94, 5.3%) harbored mutations with MSAF > 10% (Figure S7A, Supporting Information), suggesting that there existed a minuscule subset that might be classified as false negatives due to the inherent heterogeneity of the tumor. However, there was no significant difference in PFS and OS between the patients with MSAF > 10% in the tumor‐informed ctDNA group and the patients who were both tumor‐informed ctDNA and tumor‐naïve ctDNA (Figure S7B,C, Supporting Information), suggesting that the influence may be minimal.

On the other hand, to infer potential false negatives caused by ultra‐low AF due to technical limitations, we developed a regression model based on 521 patients with NSCLC in the TRACERx.^[^ ^12^, ^28^ ^]^ studies to estimate detectable MSAF and 95% confidence interval (CI) based on tumor histology and tumor size (Figure S8A,B, Supporting Information). We then estimated the lower detection limit of 95% CI of MSAF in the 94 ctDNA‐negative NSCLC cases in our study, categorizing these tumors as ctDNA non‐shedders (50 out of 94 cases) or potential technical negatives (44 out of 94 cases; Figure S8C, Supporting Information). As a result, patients with ctDNA non‐shedders or potential technical negatives showed consistently longer PFS and OS in both the ICI‐chemo arm and chemotherapy arm, compared with patients with ctDNA shedders (Figure S8D,E, Supporting Information). Furthermore, in both the tumor‐informed ctDNA‐non‐shedder or in the ctDNA potential technical negatives populations, there was no significant difference in PFS and OS between the ICI‐chemo and chemotherapy arms (Figure S8F–K, Supporting Information).

These results suggest that even though false negatives due to ultra‐low AF may exist in a very small proportion of patients, the predictive value of a tumor‐informed negative ctDNA result for overall prognosis and treatment choice remains unaffected.

### 2.7 Dynamic Change of Tumor‐Informed ctDNA Status Predicts PFS and OS as a Complementary Method to Imaging Evaluation

Patients with on‐treatment ctDNA clearance have shown improved long‐term outcomes compared with those with persistent positivity.^[^ ^10^, ^11^ ^]^ Therefore, we examined patients with baseline and on‐treatment plasma samples to explore whether dynamic changes in tumor‐informed ctDNA can predict therapeutic responsiveness.

**Figure 5:**
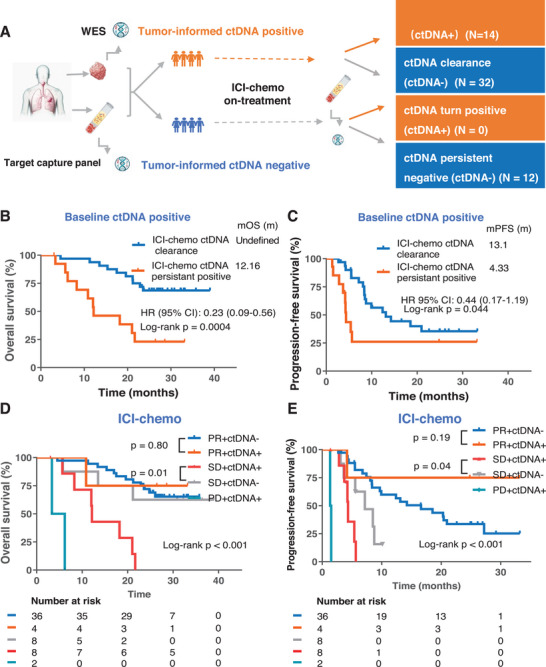
Dynamic change of tumor‐informed ctDNA status predicts PFS and OS as a complementary method to imaging. A. Schema of analysis. B‐C. Kaplan–Meier curves comparing overall survival (OS) and progression‐free survival (PFS) between different ctDNA dynamic change groups among patients who were treated with ICI‐chemo in the tumor‐informed ctDNA positive group. The HRs, corresponding 95% CIs, and statistical significance of the difference between the groups were computed using the Cox proportional hazards model. D‐E. Kaplan–Meier curves comparing overall survival (OS) and progression‐free survival (PFS) in patients who were treated with ICI‐chemo stratified by radiographic response in on‐treatment ctDNA‐negative (baseline negative or turned negative at C3D1) and on‐treatment ctDNA‐positive (baseline positive or turned positive at C3D1) populations. P values were calculated by the log‐rank test.

After two cycles of treatment (6 weeks post‐treatment), on‐treatment plasma samples (day 1 of the 3rd cycling, C3D1) were available for 91 patients, including 58 patients who received ICI‐chemo and 33 patients who received chemotherapy. The OS of these 91 patients was comparable to that of the BEP and ITT populations (Figure S9A, Supporting Information). ctDNA clearance was defined as the switch from ctDNA‐positive (at least one tissue‐derived mutation detected at baseline) to ctDNA‐negative (no tissue‐derived mutations detected at C3D1). For the patients who received ICI‐chemo, those with ctDNA clearance exhibited longer PFS and OS compared with those who remained ctDNA positive at C3D1 (log‐rank p = 0.0004 for OS and p = 0.044 for PFS; **Figure** **5**B,C). However, this trend was not significant in the chemotherapy arm, possibly due to the limited sample size (log‐rank p = 0.18 for PFS and p = 0.44 for OS; Figure S9B,C, Supporting Information).

To determine whether ctDNA testing can serve as a complementary method to imaging for predicting OS and PFS in ICI‐chemo, we further analyzed the difference between on‐treatment ctDNA+ (ctDNA persistent‐positive and ctDNA‐turn‐positive) and on‐treatment ctDNA‐ (ctDNA clearance and ctDNA persistent‐negative) patients stratified by clinical response (PR, PD, and SD). The results showed that even in patients with radiographic assessment of SD, those with on‐treatment ctDNA could predict a longer PFS and OS compared to those with on‐treatment ctDNA+ (log‐rank p < 0.05; Figure 5D,E). The difference in survival benefit was not statistically significant in patients with PR, likely due to the relatively small sample size (Figure 5D,E).

These results suggest that dynamic ctDNA status may complement radiographic assessment to predict PFS and OS.

### 2.8 Validation of Tumor‐Informed ctDNA Status in Predicting Superior Outcomes of ICI‐Chemo Over Chemotherapy

RATIONALE 304 and RATIONALE 307 were used to further validate the predictive role of tumor‐informed ctDNA status. RATIONALE 304 and RATIONALE 307 were designed to demonstrate the PFS superiority with first‐line PD‐1 antibody versus chemotherapy in advanced non‐squamous non‐small cell lung cancer (non‐LUSC) and squamous non‐small cell lung cancer (LUSC), respectively. Both trials were conducted by the same pharmaceutical company in collaboration with the same principal clinical investigators, ensuring consistency in treatment efficacy assessments. These trials were merged into a single validation cohort to ensure that the patients included in the analysis represented all pathological subtypes of NSCLC. The sequencing methods, platforms, and definitions of tumor‐informed ctDNA were consistent with those used in the CHOICE‐01 study (see **Methods**). Patients with both tissue and plasma sequencing data were considered as BEP. The BEP for tumor‐informed ctDNA analysis included 82 and 62 patients in the RATIONALE 304 and RATIONALE 307 trials, respectively. The baseline characteristics and efficacy profiles of BEP were comparable to those of the ITT (Table S9). PFS in the BEP and ITT populations was also comparable (Figure S10A,B, Supporting Information). The baseline characteristics were balanced between the ICI‐chemo and chemotherapy groups in the BEP (*Chi‐square test*, p > 0.05).

**Figure 6:**
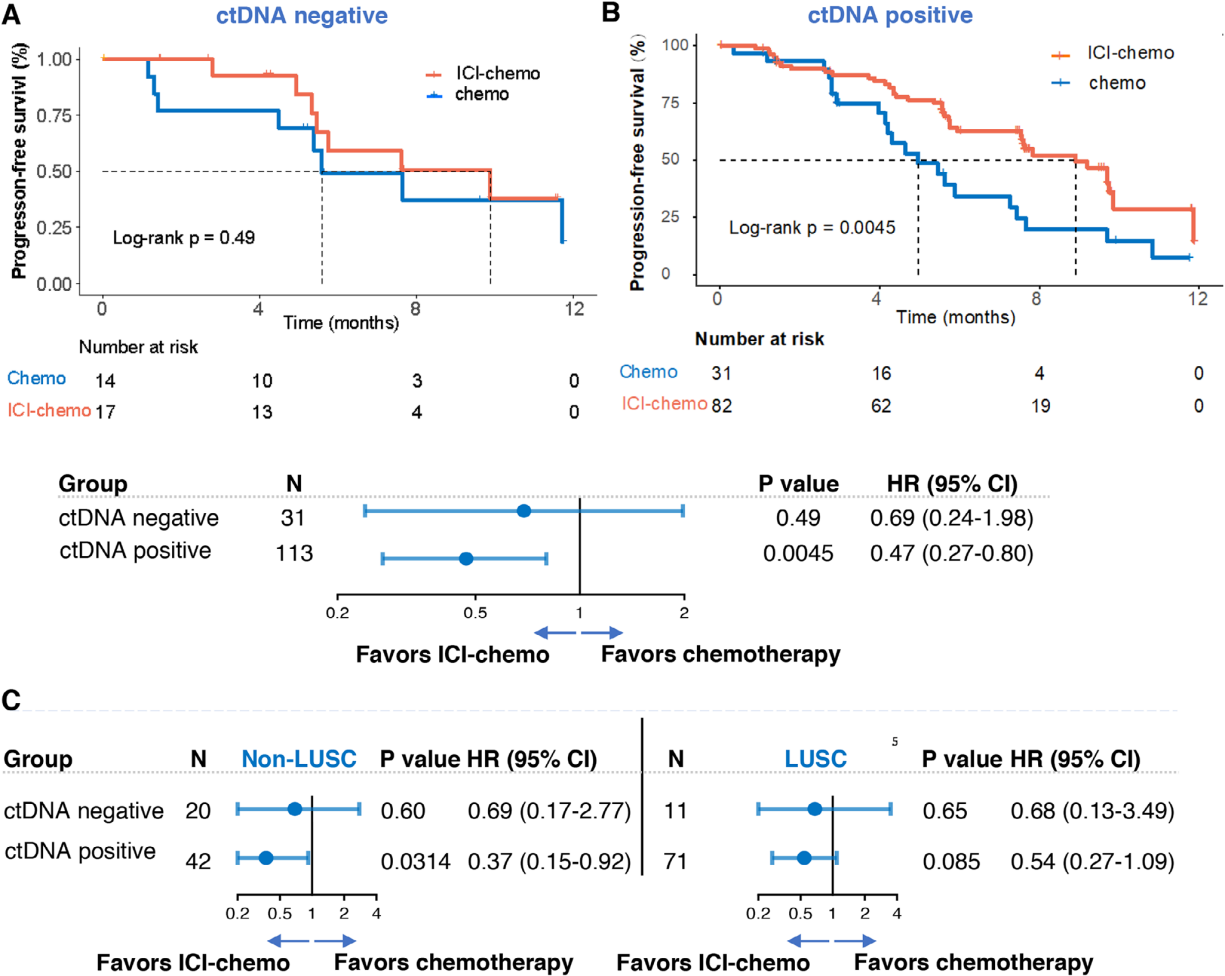
Predictive role of tumor‐informed ctDNA in the RATIONALE‐304/307 cohort. A‐B. Kaplan–Meier curves comparing progression‐free survival (PFS) among patients who were treated with ICI‐chemo versus chemotherapy in patients with tumor‐informed ctDNA+ or tumor‐informed ctDNA‐. Upper, Kaplan–Meier curves depicting the treatment comparison; lower, forest plot depicting the relative efficacy of chemo‐ICI versus chemotherapy. The HRs, corresponding 95% CIs, and statistical significance of the difference between the groups were computed using the Cox proportional hazards model. C. Forest plot depicting the comparison of ICI‐chemo and chemotherapy in tumor‐informed ctDNA+ and tumor‐informed ctDNA‐ groups, non‐lung squamous cell carcinoma (non‐LUSC) and lung squamous cell carcinoma (LUSC), respectively. The HRs, corresponding 95% CIs, and statistical significance of the difference between the groups were computed using the Cox proportional hazards model.

Consistent with previous findings, patients who were tumor‐informed ctDNA+ had improved PFS with ICI‐chemo compared to those who received chemotherapy (HR = 0.47; 95% CI: 0.27–0.80; p = 0.0045; **Figure** **6**A). No significant difference in PFS was observed between treatment arms for patients with tumor‐informed ctDNA‐ (HR = 0.69; 95% CI: 0.24‐1.98; p = 0.49; Figure 6B). The predictive role of tumor‐informed ctDNA for ICI‐chemo was consistent across both non‐LUSC (RATIONALE 304) and LUSC (RATIONALE 307) cohorts (Figure 6C,D; Figure S10C–E, Supporting Information).

These findings suggest that tumor‐informed ctDNA is a promising biomarker for predicting the superiority of ICI‐chemo over chemotherapy in first‐line advanced NSCLC.

## 3 Discussion

In this study, we demonstrate that tumor‐informed ctDNA status can effectively and conveniently differentiate the patient population suitable for treatment with ICI‐chemo combination rather than chemotherapy alone in the first‐line setting. Additionally, its predictive ability is independent of established immune biomarkers and surpasses other ctDNA metrics. In addition to radiographic assessment, the clearance of ctDNA can also serve as an indicator of the efficacy of the ICI‐chemo regimen. Our results demonstrate for the first time that tumor‐informed ctDNA status may provide a valuable strategy for guiding first‐line ICI‐chemo treatment in advanced‐stage NSCLC.

In our study, patients with tumor‐informed ctDNA+ showed better PFS and OS when treated with ICI‐chemo compared to those receiving chemotherapy alone. However, for patients with tumor‐informed ctDNA‐, ICI‐chemo yielded PFS and OS outcomes comparable to chemotherapy. These findings suggest that ICI‐chemo may confer greater clinical benefit in ctDNA‐positive patients, whereas chemotherapy alone might be sufficient for those who are ctDNA‐negative at baseline. Moreover, the predictive role of tumor‐informed ctDNA for PFS remained after adjusting for other clinical variables, including histology, smoking status, PD‐L1, and TMB. Given the increased risk of immune‐related adverse events and the higher costs associated with immunotherapy, chemotherapy may represent a more appropriate option for ctDNA‐negative patients. Early stratification based on tumor‐informed ctDNA status may thus help optimize treatment decisions, avoiding overtreatment in patients less likely to benefit from ICI‐based combinations.^[^ ^13^, ^14^ ^]^ However, our ctDNA‐negative subgroup had a relatively small sample size, limiting the statistical power to formally establish the non‐inferiority of chemotherapy. These preliminary findings provide a rationale for future non‐inferiority trials evaluating treatment de‐escalation strategies guided by ctDNA status. Finally, exploratory genomic analyses revealed alterations in the ERBB, mTOR, and RAS signaling pathways among ctDNA‐negative patients, highlighting potential avenues for future combinatorial strategies in this subgroup.

ctDNA release is not only associated with tumor characteristics and shedding kinetics but is also linked to increased immunogenicity.^[^ ^29^, ^30^ ^]^ In our study, we observed that patients with tumor‐informed ctDNA+ at baseline exhibited higher neoantigen levels and chromosomal instability, represented by higher LOH, wGII, and ploidy, compared to tumor‐informed ctDNA‐ patients. These results are consistent with previous studies. Thomas et al. reported that inflammatory response signaling was significantly upregulated in ctDNA shedders compared to ctDNA non‐shedders.^[^ ^13^ ^]^ Similarly, Christopher et al reported a higher wGII, LOH, and a higher percentage whole genome doubling (WGD) in tumor‐informed ctDNA‐positive patients.^[^ ^28^ ^]^ These studies suggest that although a high ctDNA burden generally indicates a poorer prognosis regardless of the therapeutic regimen, patients with detectable ctDNA are more likely to benefit from the addition of ICI therapy compared to chemotherapy alone, likely due to the enhanced immune response in ctDNA‐positive patients.

In our study, the predictive role of tumor‐informed ctDNA for ICI‐chemo was not confounded by other immune‐related biomarkers such as TMB, PD‐L1 expression, and neoantigen levels. Our findings highlight that tumor‐informed ctDNA status may provide complementary predictive information beyond PD‐L1 expression in guiding first‐line ICI‐chemo decisions for advanced NSCLC. While PD‐L1 remains an established biomarker for anti‐PD‐1 monotherapy, particularly in patients with PD‐L1 ≥50%, our data suggest that ctDNA status can help identify patients who are more likely to benefit from ICI‐chemo regardless of PD‐L1 expression. Thus, for patients with low PD‐L1 expression but positive ctDNA, ICI‐chemo may be preferred. However, whether patients with high PD‐L1 expression but negative ctDNA should receive ICI monotherapy instead of chemotherapy or ICI‐chemo needs further investigation. Moreover, the predictive role of tumor‐informed ctDNA+ for the superiority of ICI‐chemo versus chemotherapy was consistent across most clinical subgroups, including age, sex, ECOG, and smoking history. Notably, patients with non‐LUSC gained significant PFS and OS benefits from ICI‐chemo in the tumor‐informed ctDNA+ group, whereas patients with LUSC obtained only additional PFS benefits. This observation is consistent with the original clinical trial,^[^ ^3^ ^]^ which might be partially explained by the higher crossover rate in this subgroup, as previously discussed.^[^ ^3^ ^]^ These observations warrant further validation in prospective trials, but they underscore the potential value of integrating ctDNA analysis into personalized treatment strategies.

We observed that among patients initially positive for ctDNA, those who achieved ctDNA clearance exhibited a better therapeutic response to ICI‐chemo, indicating that the dynamic change of ctDNA can serve as a biomarker for treatment efficacy. This finding underscores the utility of incorporating ctDNA monitoring in the clinical application of ICI‐chemo to better gauge treatment outcomes. Patients who maintained a negative ctDNA status post‐treatment experienced sustained benefits from ICI‐chemo. By contrast, those who remained ctDNA‐positive at both baseline and during therapy were less likely to derive significant clinical benefit from ongoing ICI‐chemo, suggesting that this group may be more suitable for exploratory clinical trials investigating intensified or alternative therapeutic strategies. Radiologic SD represents a particularly challenging clinical category, encompassing patients with slowly progressive disease, indolent non‐responding disease, and radiologically subtle responses. This classification is inadequate to accurately identify patients who may benefit from ICI‐chemo. In our study, tumor‐informed ctDNA retained the predictive value in a subset of patients who demonstrated SD based on radiographic assessments. These results indicate that tumor‐informed ctDNA may offer complementary prognostic information to traditional imaging techniques, potentially enhancing the accuracy of therapeutic efficacy assessment.

The sensitivity of ctDNA detection in NSCLC exhibits significant variability, even in advanced stages, with detection rates ranging from 60% to 85%.^[^ ^9^, ^31^, ^32^, ^33^, ^34^, ^35^ ^]^ This variability suggests that a proportion of late‐stage NSCLC tumors are ctDNA low shedders. Additionally, NSCLC is particularly susceptible to interference from CH, with CH mutations potentially mistaken for tumor‐derived ctDNA, thereby complicating the interpretation of ctDNA results.^[^ ^22^, ^36^, ^37^ ^]^ In this study, we first investigated the predictive role of tumor‐naïve ctDNA for superior survival with ICI‐chemo over chemotherapy. However, it failed to predict the superior efficacy of ICI‐chemo over chemotherapy. The FDA‐led Sequencing Quality Control Phase 2 (SEQC2) project reported that when the variant allele frequency was below 0.5%, ctDNA detection became unreliable and varied widely between assays.^[^ ^38^ ^]^ Methodologically, the detection of tumor‐informed ctDNA significantly lowers the LOD for plasma ctDNA, thereby increasing the sensitivity of ctDNA detection.^[^ ^12^ ^]^ In our study, 13 (4.3%) patients with an MSAF < 0. 5% (under the LOD of the sequencing platform) were classified as tumor‐informed ctDNA positive, and tumor‐informed ctDNA could detect AF as low as 0.07%. In terms of specificity, the tumor‐informed approach also identified 35 (37.2%) ctDNA‐negative patients who were ctDNA‐positive, using the tumor‐naïve approach. The survival of these patients was not different from that of patients with both tumor‐informed and tumor naïve negative ctDNA. Moreover, there was no significant difference in survival between the ICI‐chemo and chemotherapy groups among these patients. In consideration that 20% (7 out of 35) of patients only harbored plasma‐specific gene mutations, and 37.1% (13 out of 35) only harbored CH‐associated gene mutations in their plasma, the tumor‐informed approach offers an opportunity to reduce the risk of mistakenly tracking germline and CH signals.

Previous investigations have underscored the influence of clinical baseline characteristics on ctDNA shedding, such as histology, tumor size, lymphatic metastasis, lymphatic vascular invasion, and smoking status in stage I‐III NSCLC.^[^ ^28^ ^]^ However, the baseline clinical characteristics that contribute to ctDNA release in advanced‐stage NSCLC remain largely unexplored. Our study provides further insights into this area, demonstrating that patients with squamous cell lung cancer, smoking history, or larger tumor sizes exhibit more ctDNA shedding, a finding that aligns with previous research.^[^ ^12^ ^]^ Additionally, we identified adjuvant treatment, *TP53* mutations, and genomic instability as other factors associated with increased ctDNA release.

One potential limitation in the analysis of tumor‐informed ctDNA is the LOD, which can lead to the missing of low‐frequency mutations. However, prior research in advanced‐stage NSCLC indicated that the mean VAF ranged from 5% to 7%.^[^ ^39^ ^]^ In our study, the mean VAF was 5.73%. This concordance suggests that the current target capture panel, with a sequencing depth of 10,000X, is sufficiently sensitive to encompass the majority of variant alleles, thereby minimizing the likelihood of false negatives attributable to inadequate LOD. Additionally, we simulated undetectable VAF in tumor‐informed ctDNA‐negative samples based on tumor size and pathology. Our findings indicated that 44 patients were negative, probably because of potential technical limitations. However, there was no significant difference between ICI‐chemo and chemotherapy treatments in the 44 patients. The predictive value of a negative tumor‐informed ctDNA result for overall prognosis and treatment choice remains unaffected. Another potential source of false negatives in the tumor‐informed approach is intratumoral heterogeneity (ITH), which is constrained by single‐region tissue sampling. Despite our comprehensive 520‐panel capturing the mutation landscape (COSMIC database) of almost all advanced‐stage NSCLC patients, potential false negatives cannot be completely ruled out because of mutation sites beyond the detection range of the 520 Panel. To address this issue, multiregion tissue sampling is necessary to unravel heterogeneity, albeit with limited clinical applicability. Notably, most tumor tissue samples used in the present study were recent biopsy specimens obtained from tumor lesions after the diagnosis of metastasis (83%). Only a small proportion consisted of archived formalin‐fixed tumor surgical specimens (17%), all of which were collected within the past two years. The observed concordance in ctDNA positivity rates between biopsy and surgical specimens showed no significant difference. Furthermore, a previous study demonstrated that the number of detectable mutations did not differ significantly between fresh‐frozen tumor biopsy tissues and archival tumor tissue.^[^ ^10^ ^]^ Therefore, the potential influence of archived tissue specimens on false negativity is likely minimal in the present study. Nevertheless, future prospective studies should further evaluate the impact of tissue source and sampling timing on the predictive value of tumor‐informed ctDNA analysis.

Our study has several limitations. First, t the sample size for tumor‐informed ctDNA‐negative patients was relatively small, limiting the statistical power to conclusively demonstrate the non‐inferiority of chemotherapy. Additionally, although the observed trend in OS was consistent with our hypothesis, the interaction p‐value was not significant, and the results should thus be interpreted with caution. Therefore, a prospective clinical trial is warranted to validate these findings and further define their clinical utility. Second, the mechanisms of tumor‐informed ctDNA predictive role for ICI‐chemo require further investigation. Third, tissue samples were subjected to WES. Implementing tumor‐informed ctDNA testing in clinical practice faces limitations related to sample availability, cost, and turnaround time. The high expense and the extended time required for WES may hinder its widespread adoption in time‐sensitive treatment decisions. In this context, a tissue sequencing panel that exactly matches this 520‐gene ctDNA panel may serve as a more practical alternative to WES, potentially shortening turnaround times and enabling earlier treatment decision‐making. Nevertheless, ongoing technological advancements aimed at reducing sequencing costs and improving efficiency will be critical to the broader clinical application of tumor‐informed ctDNA strategies.

## 4 Conclusion 

Our findings underscore the potential of tumor‐informed ctDNA status as a predictive biomarker for superior survival of ICI‐chemo over chemotherapy in advanced NSCLC. Furthermore, ctDNA clearance may serve as an indicator of the efficacy of ICI‐chemo beyond radiographic imaging assessment. These results may potentially improve the management of advanced NSCLC.

## 5 Experimental Section

### Patients and Study Design

The CHOICE‐01 study (ClinicalTrials.gov number NCT03856411), a multicenter, randomized, double‐blind, placebo‐controlled, phase III trial, has been previously reported (Figure S1, Supporting Information).^[^ ^3^ ^]^ The protocol and statistical analysis plan for CHOICE‐01, approved by the ethics committees at each site, adhered to the International Conference on Harmonization Good Clinical Practice guidelines and the principles of the Declaration of Helsinki. Written informed consent was obtained from all participating patients. All patients provided informed consent, and this study received approval from the ethics committees of the National Cancer Center (NCC18‐222/1780, 18–023/1624, 18–024/1625).

In BEP, tissue samples and ctDNA were collected prospectively and measured using WES and a 520‐gene target capture sequencing panel, respectively (Table S10). WES was conducted on the tumors and matched normal samples to identify patient‐specific mutations. The presence of one or more of these mutations in plasma was defined as ctDNA+ (Figure S1A, Supporting Information).

PD‐L1 expression was assessed via immunohistochemical staining with JS311 antibody using a validated assay in a central laboratory (MEDx, Suzhou, China). A cross‐correlation study^[^ ^3^ ^]^ revealed similar PD‐L1 staining patterns and scores with JS311, 22C3, 28–8, and SP263 antibodies in biopsy samples from patients with NSCLC. PD‐L1 positivity was defined as a tumor cell (TC) expression ≥1%.

### Whole‐Exome Sequencing

In accordance with a previous study,^[^ ^3^ ^]^ we performed WES on formalin‐fixed paraffin‐embedded (FFPE) tumor samples. A pathologist reviewed all FFPE samples to confirm that each sample had nucleated cellularity >80% and no tumor content <20%. These specimens were obtained from patients with advanced NSCLC enrolled in the CHOICE‐01 study (Figure 1A) before the initiation of any treatment. Patients with a history of second primary tumors were excluded from the study. All tissue samples intended for WES sequencing consist of recent biopsy tissue from tumor lesions obtained after the diagnosis of metastasis83.0% or archived formalin‐fixed tumor surgical tissue specimens (17.0%); For archived specimens, the samples should be collected within the past two years. All tissue samples for WES analyses were subjected to rigorous quality control measures, and any sample failing to meet these standards was excluded from downstream analyses. The central lab (OrigiMed, Shanghai, China) was responsible for DNA extraction, amplification, quality control, and sequencing of tumor biopsies and their matched peripheral blood mononuclear cells (PBMCs) samples. The mutation calling, variant filter criteria, annotation process, and quality control were described previously,^[^ ^3^ ^]^ and germline variants with a mutation rate >3% were filtered out. The target coverage for FFPE tumor slides and matched PBMC samples is ≈500 × and 150 ×, respectively.^[^ ^3^ ^]^ The specifics of the DNA library preparation and sequencing have been documented elsewhere. Raw sequence data were processed using a customized analysis pipeline as previously reported.^[^ ^3^ ^]^ The assessment of genomic alterations included microsatellite stability status, single‐base substitutions, short and long insertions/deletions (INDELs), copy‐number variants, gene rearrangements, and fusions.

### DNA Extraction and Targeted Sequencing of Plasma Samples

Plasma samples were prospectively collected at baseline (day 1at 1^st^ cycling, C1D1) and 6 weeks after randomization (day 1 at 3rd cycling, C3D1) and tested with a targeted panel of 520 cancer‐related genes (OncoScreen Plus®, covering a 1.86 MB region of the human genome).

DNA extraction and targeted sequencing were performed at Burning Rock Biotech, a commercial clinical laboratory that has demonstrated impressive performance in the FDA‐led Sequencing Quality Control Phase 2 (SEQC2) liquid biopsy program.^[^ ^38^ ^]^ To elaborate, 8 mL of venous blood was drawn from patients, and centrifugation at 2000 × g for 10 min at room temperature within 2 h of collection effectively separated peripheral white blood cells (WBCs) and plasma. The supernatant plasma was then transferred to a new tube and subjected to a second centrifugation at 3000 rpm for 10 min at room temperature, collected as plasma samples, and stored at −80 °C. PBMCs, which are a subset of WBCs, were isolated from peripheral blood by layering diluted blood samples with 100% Ficoll in a centrifuge tube. Centrifugation to separate the mononuclear cell layer was used for genomic DNA extraction, serving as germline controls. cfDNA from plasma samples was extracted using the QIAamp® Circulating Nucleic Acid Kit (Qiagen, Hilden, Germany) for manual extraction, and genomic DNA (gDNA) from WBCs (which are the PBMCs mentioned in the previous paragraph) was extracted using the MagPure FFPE DNA/RNA LQ Kit (Magen Biotech, Guangzhou, China), following the standard protocol provided by the manufacturer (Qiagen, CA, USA). DNA quantification, library construction, sequencing, and data processing were performed as previously described.^[^ ^40^, ^41^ ^]^ Briefly, for each sample, at least 50 ng of DNA was fragmented and subjected to end repair, phosphorylation, dA addition, and adaptor ligation for library construction. Next, the DNA library consisting of fragments of sheared genomic DNA and cfDNA ranging from 200 to 400 bp was purified using the Agencourt AMPure XP Kit (Beckman Coulter, CA, USA), hybridized with capture probe baits, selected with magnetic beads, and subsequently amplified. Capture‐based targeted sequencing was performed using a commercial panel, OncoScreen Plus® (Burning Rock Biotech). Inc, which consists of 520 genes and spans 1.86 megabases of the human genome (Table S8) on the Novaseq 6000 platform (Illumina, Inc., San Diego, CA, USA) with paired‐end reads at a target average sequencing depth of 10 000× for plasma cfDNA and 2000 × for WBC gDNA. Raw sequencing data were trimmed and mapped onto the human genome (hg19). Variant annotation and calling were performed using the Genome Analysis Tool Kit (GATK) version 3.2,^[^ ^42^ ^]^ VarScan version 2.4.3^[^ ^43^ ^]^ and VarDict version 1.5.1.^[^ ^44^ ^]^ Matched PBMCs were generated using the same methodology as described for ctDNA variant calling. After variant calling and annotation, mutations were filtered against common single‐nucleotide polymorphisms (SNPs) found in the 1000 Genomes ExAC, dbSNP, ESP6500SI‐V2, and ClinVar databases. SNVs and Indes required support from at least 8 and 2 supporting reads, respectively. Reads with mapping quality of < 60 and > 8 mismatches were removed.

Alterations classified as positive in PBMCs were presumed to be germline/CHIP and were excluded from the analyses. ctDNA was quantified in units of mean tumor molecule per milliliter plasma (MTM/mL).

### Analytical Validation Methods

In this study, we determined the LOD for 520 gene liquid biopsies (OncoScreen Plus®) for blood testing. We employed a gradient dilution of mutations defined by SeraCare's NGS reference standards (Material Number: 0710–0528, Lot #: 10509970) with a 5% minor allele frequency (MAF), encompassing a series of SNVs and INDELs calibrated via PCR using the BioRad QX200™ Droplet Digital™ PCR System. At a sequencing depth of 10 000 ×, we assessed the gradient‐diluted samples at MAF levels of 5%, 1%, 0.5%, 0.25%, and 0.1%. Binomial distribution probability analysis revealed that the lowest detectable abundance at the 95% confidence level was 0.208% for SNVs and 0.29% for INDELs. Consequently, the LOD for detecting individual SNVs and INDELs using this panel was established to be 0.5%.

### TMB and Neoantigen Analysis

TMB was calculated as the total number of non‐synonymous mutations in each sample. Potential neoantigens were identified by running POLYSOLVER (v1.0)^[^ ^45^ ^]^ to identify MHC Class I alleles from matched normal WES data. The predicted binding affinity for all possible 9mer and 10mer peptide sequences overlapping single‐ and di‐nucleotide somatic variants was assessed using NetMHCPan‐4.0.^[^ ^46^, ^47^ ^]^ Neoantigens with percentile ranks of two or less for any Class I allele in the same patient were counted as predicted binders.

### Chromosomal Instability Analyses

Copy‐number data, including allele‐specific copy number, LOH, purity, and ploidy estimates, were derived from the WES pipeline. Segment‐level copy number alterations (CNAs) were identified using the Sequenza R package (v3.0.0).^[^ ^48^ ^]^ and gene‐level CNAs were identified using GISTIC (v.2.0).^[^ ^49^ ^]^ from the segment‐level sequenza results. wGII was calculated as the fraction of SNPs across the genome present at an aberrant copy number relative to the total genome of the sample.^[^ ^50^ ^]^ The homologous recombination deficiency‐based loss of heterozygosity (HRD‐LOH) score is 15 Mb, exceeding LOH regions that do not cover the whole chromosome in a sample.^[^ ^51^ ^]^ To determine the HRD‐LOH score using WES data, binary Alignment/Map format (BAM) files of tumor and normal samples were applied to the allele‐specific copy number analysis of tumors (ASCAT) algorithm.^[^ ^52^ ^]^ Tumor purity and ploidy were identified using the FACETS R package (v.0.6.2).^[^ ^53^ ^]^ run with default parameters. For each copy number segment identified, FACETS provides allele‐specific estimations of copy numbers, namely, the lower copy number (LCN) and total copy number (TCN).

### Identifying Probable Technical Negative and Low‐Shedding NSCLC

We generated a linear regression model in which log10‐transformed tumor size and histology were used to predict log10‐transformed MSAF of ctDNA in 521 NSCLC patients in the TRACERx study.^[^ ^12^, ^28^ ^]^ We used this model to predict MSAF and lower the 95% CI of MSAF in 94 NSCLC that were negative for ctDNA in the CHOICE‐01 study. Using the above linear model, we classified cases as possible technical negatives if the lower 95% CI for the predicted MSAF of ctDNA was below 0.1% (5 fold lower than the LOD) and as possible low‐shedders if the lower 95% CI for the predicted MSAF of ctDNA was above 0.1%.

### Study Design and Participants of Rationale 304/ Rationale 307 Study

This retrospective study utilized a combination of data from two open‐label, multicenter, randomized, phase III studies, RATIONALE‐304 (NCT01903993)^[^ ^25^ ^]^ and RATIONALE‐307 (NCT02008227).^[^ ^26^ ^]^ RATIONALE 304 and RATIONALE 307 were designed to demonstrate the superiority of PFS with first‐line tislelizumab plus chemotherapy versus chemotherapy alone in advanced non‐squamous non‐small cell lung cancer (non‐squamous‐NSCLC) and squamous non‐small cell lung cancer (squamous‐NSCLC), respectively. Both studies met their primary objectives. In each trial, patients were randomized in a 1:1 ratio to receive either tislelizumab plus chemotherapy or chemotherapy alone. Comprehensive descriptions of the eligibility criteria and recruitment methodologies for both trials have been previously published.^[^ ^25^, ^26^ ^]^ The study adhered to the Declaration of Helsinki and was approved by the local ethics committees. The RATIONALE 304 and RATIONALE 307 trials included 334 and 355 patients in the primary intention‐to‐treat population, respectively.

DNA panel sequencing was conducted on baseline tumor specimens and plasma samples collected before the first treatment, as previously described.^[^ ^54^ ^]^ The procedure for blood collection and processing was the same as that used in the CHOICE‐01 study. Capture‐based targeted sequencing was performed using a commercial panel, OncoScreen Plus® (Burning Rock Biotech). Inc., which is similar to the CHOICE‐01 study. Mutation calling, variant filter criteria, annotation process, and quality control were described previously for tissue.^[^ ^54^ ^]^ For plasma DNA sequencing, mutation calling, variant filter criteria, annotation process, and quality control were consistent with CHOICE‐01 plasma DNA sequencing. Positive tumor‐informed ctDNA were defined as one or more tissue‐derived variants that appeared in the plasma.

### Statistical Analysis

Continuous variables were compared using the two‐sided Mann‐Whitney U test, and categorical variables were compared using the chi‐square test or Fisher's exact test, as appropriate. Continuous variables are presented as mean ± standard deviation (SD) if approximately normally distributed, or median (interquartile range, IQR) if non‐normally distributed. Categorical variables are presented as n (%). No data transformation or imputation was performed; analyses were based on complete cases.

Survival probabilities were estimated using the Kaplan‐Meier method and compared by the log‐rank test. Hazard ratios (HRs) with 95% confidence intervals (CIs) and corresponding p values were estimated using Cox proportional hazards models. Biomarkers of differential treatment effect—including tumor‐informed ctDNA, MTM, bTMB, and MSAF—were assessed by including an interaction term between the biomarker and treatment arm in Cox models (models included terms for biomarker, treatment arm, and their interaction). The significance of the interaction term was used to infer the differential treatment effect.

All statistical tests were two‐sided, and a significance level of α = 0.05 was used. Where multiple comparisons were performed, p‐values were adjusted using the Benjamini‐Hochberg false discovery rate. Statistical analyses were performed using R version 4.3.0 and GraphPad Prism 8.

## Conflict of interest

The authors declare no potential conflicts of interest, except the employment of Chengcheng Li, Wenchuan Xie, Guoqiang Wang, Yu Xu, and Shangli Cai in Burning Rock Biotech.

## Author Contributions

K.F., J.Z., J.X., J.D., C.L., and W.X. contributed equally to this work. S.C., Z.W., and J.W. were responsible for the conceptualization, supervision, and funding acquisition. K.F., J.X., C.L., J.Z., W.X., G.W., Y.X., B.S., R.W., J.D., H.B., and J.Z. conducted the methodology, formal analysis, visualization, and writing of the original draft. All authors contributed to the investigation, data curation, and writing—review and editing.

## Supporting information



Supporting Information

Supplemental Table 1

## Data Availability

The authors declare that the relevant data supporting the findings of this study are available within the paper and its supplementary files. The detailed sequencing data have been uploaded to the National Genomics Data Center (https://ngdc.cncb.ac.cn/bioproject/browse/PRJCA024983). Readers can contact the corresponding authors to access the patient‐level data for non‐commercial purposes. All data generated in this study are provided in the Source Data file. The remaining data are available within the Article, Supplementary, or Source Data file. Source data are provided with this paper.
