# Supplementary material for: Tumor‐Informed ctDNA in Guiding First‐Line Immunochemotherapy in Advanced Non‐Small Cell Lung Cancers
Source: Adv Sci (Weinh). 2025 Nov 25;12(48):e06565. doi: 10.1002/advs.202506565 (PMC12752622; doi:10.1002/advs.202506565)
Supplement: Supplementary file 1 — Supporting Information [file ADVS-12-e06565-s001.docx]

**Online Supplementary Material**

Fei et al. Tumor-informed ctDNA in guiding first-line immunochemotherapy in advanced non-small cell lung cancers

**Contents:**

**Supplementary Fig. 1-10**

**Figure titles and legends**

**
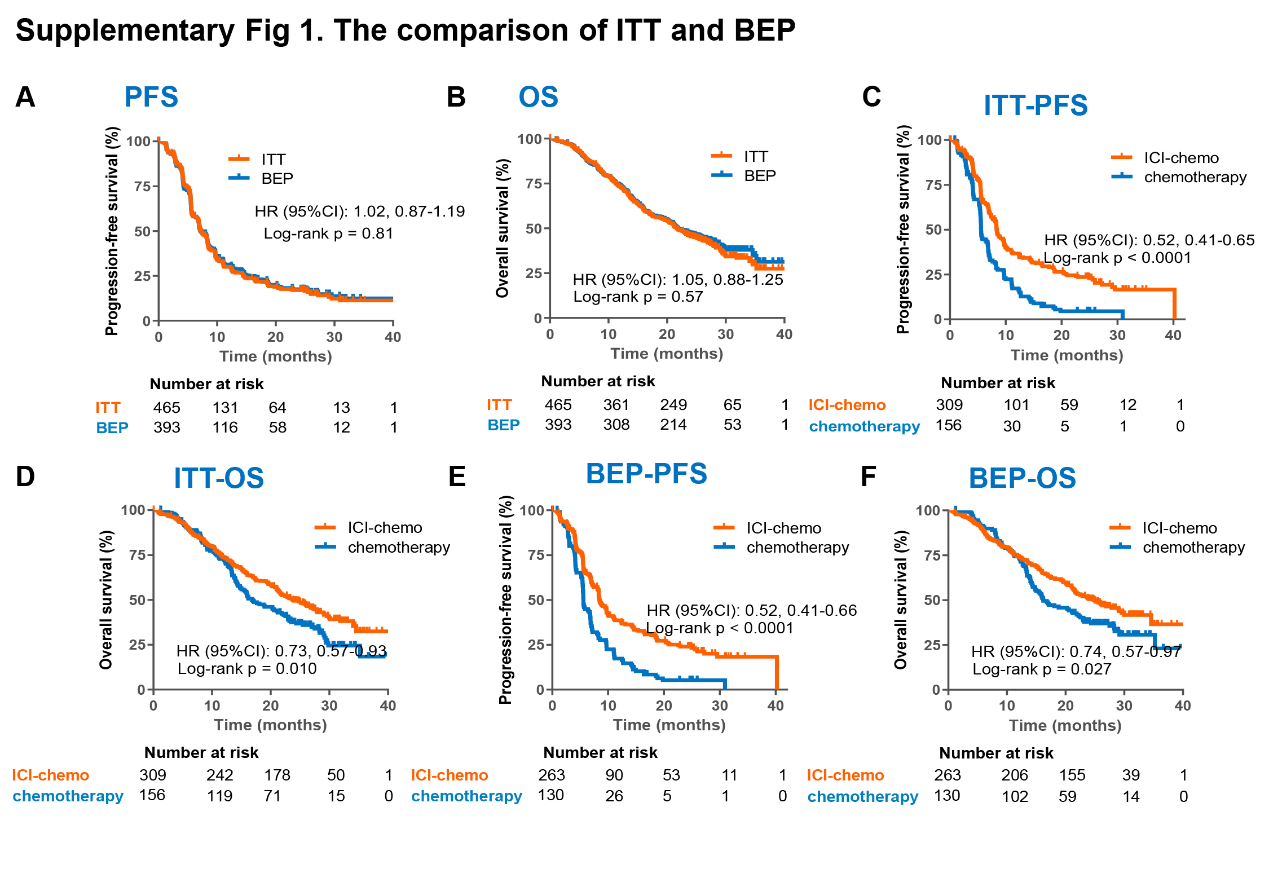
**

**Supplement Fig. 1. The comparison between BEP and IIT**

**A-B.** Kaplan-Meier curves comparing progression-free survival (PFS) and overall survival (OS) between the Intent-to-Treat (ITT) population and the biomarker evaluable population (BEP).

**C-D.** Kaplan-Meier curves of progression-free survival (PFS) and overall survival (OS) for patients treated with ICI-chemotherapy versus chemotherapy in the Intent-to-Treat (ITT) population

**E-F.** Kaplan-Meier curves of progression-free survival (PFS) and overall survival (OS) for patients treated with ICI-chemotherapy versus chemotherapy in the biomarker evaluable population (BEP). Hazard ratios (HRs) and corresponding 95% confidence intervals (CIs) were estimated using the Cox proportional hazards model. P values for survival differences were calculated using the two-sided log-rank test.

**
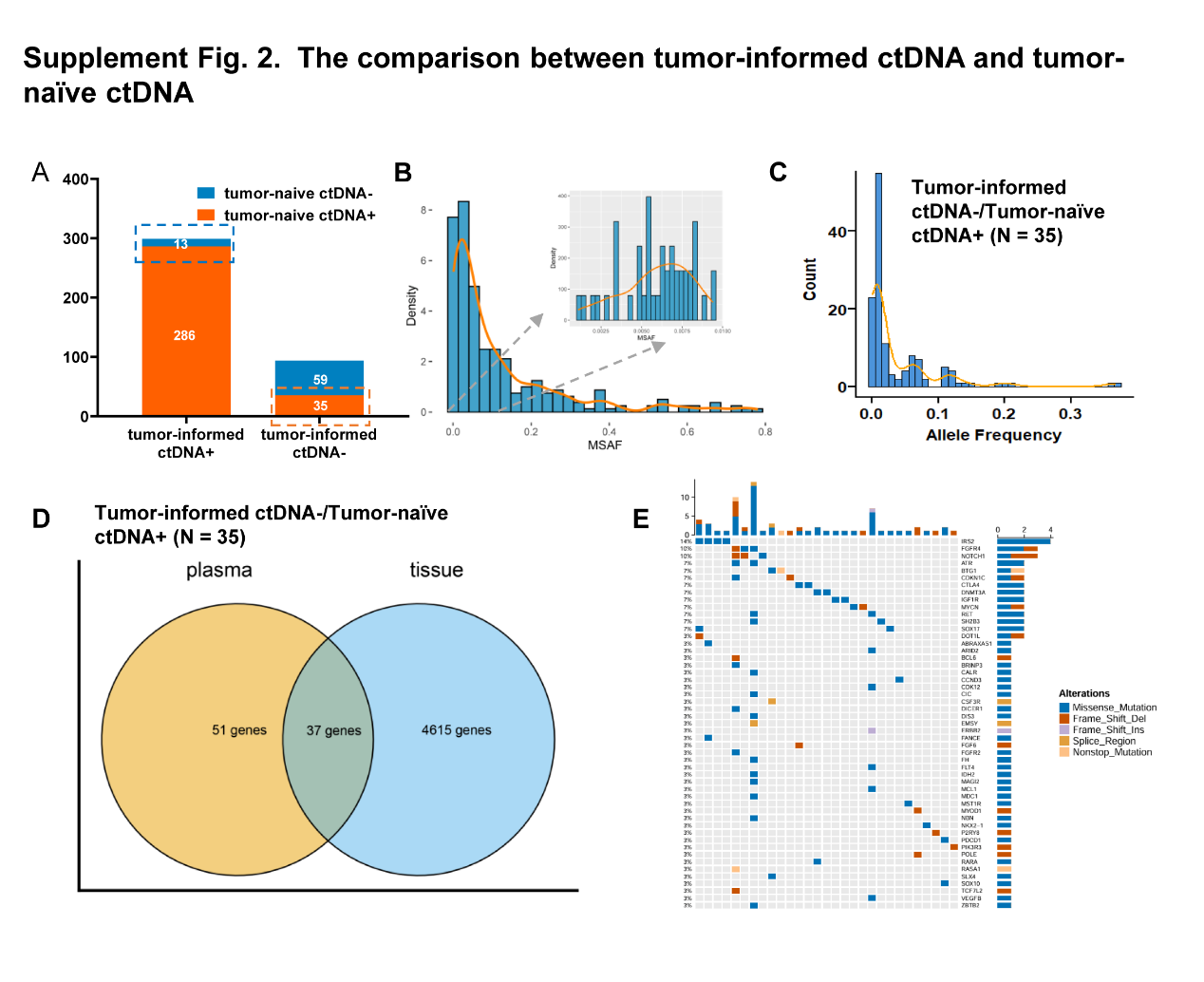
**

**Supplement Fig. 2. The comparison between tumor-informed ctDNA and tumor-naïve ctDNA**

**A.** Bar chart illustrating the overlap of positive and negative results between tumor-informed ctDNA and tumor-naive ctDNA status.

**B.** Density histogram illustrating the distribution of max somatic allele frequency (MSAF) in the tumor-informed ctDNA positive group and a smaller density histogram depicting the distribution of MSAF from 0 to 0.010.

**C.** Density histogram illustrating the distribution of allele frequency (AF) in the tumor-informed ctDNA positive/ tumor-naïve ctDNA positive group

**D.** Venn diagram illustrating the overlap of mutations derived from blood or tissue in 35 tumor-informed ctDNA-/tumor-naïve ctDNA+ patients.

**E.** Oncoprint displaying 51 plasma-specific genes.

**
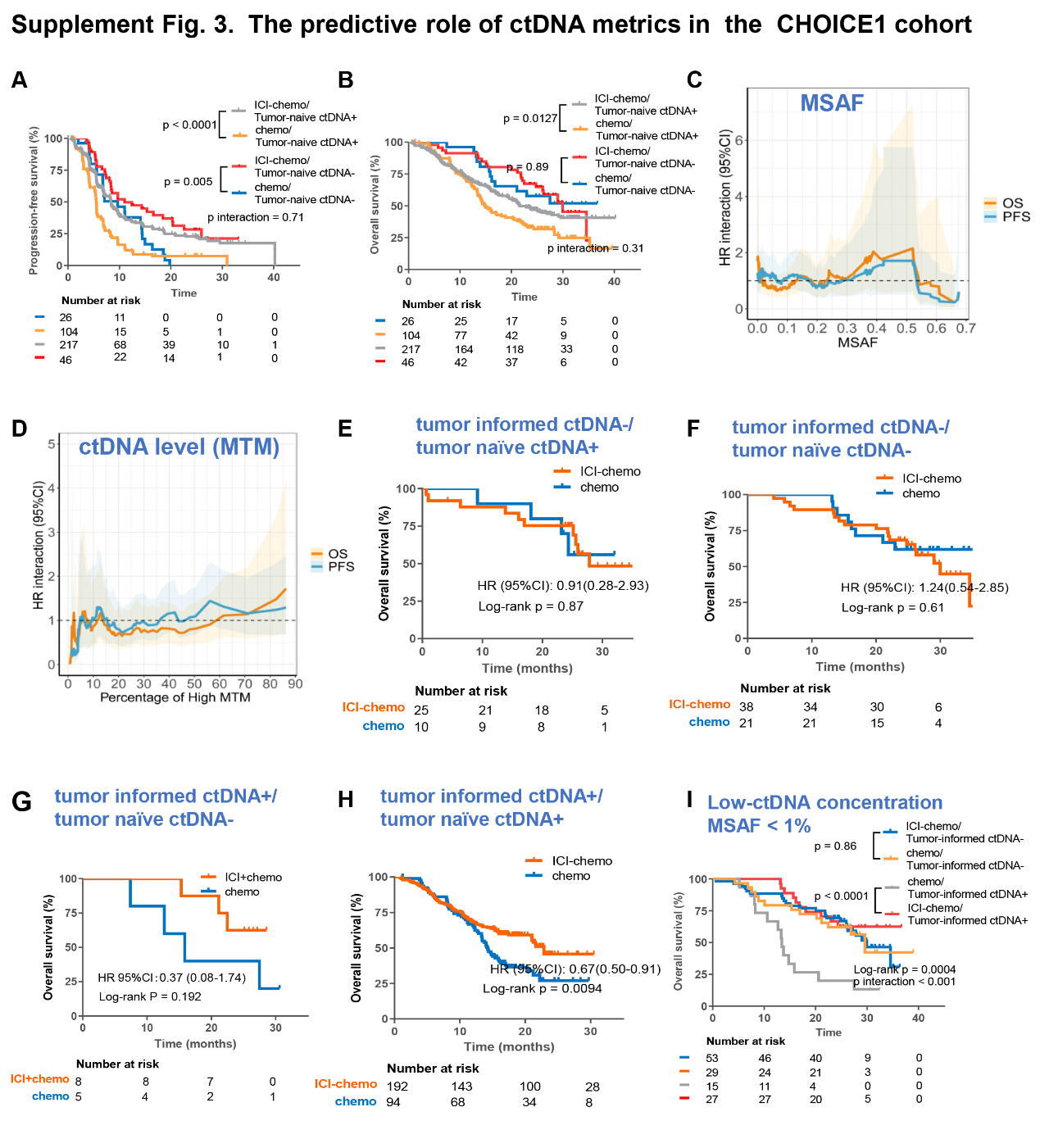
Supplementary Fig. 3. The predictive role of ctDNA metrics in the CHOICE1 cohort**

**A-B.** Kaplan–Meier estimates of Progression-Free Survival (PFS) and Overall Survival (OS) for patients stratified by tumour-naive ctDNA status and treatment.

**C.** The interaction hazard ratio (HR) between ICI-chemo and chemotherapy regarding PFS and OS, stratified by different cutoffs of max somatic allele frequency (MSAF).

**D.** The interaction HR of ICI-chemo versus chemotherapy regarding PFS and OS when stratified by different cutoffs of ctDNA level (MTM) (any available cutoff ranging from the 10th to the 90th percentile).

**E.** Kaplan–Meier curve comparing the OS of patients treated with chemotherapy versus patients treated with ICI-chemo in the tumor-informed ctDNA-/ tumor-naïve ctDNA+ group

**F.** Kaplan–Meier curve comparing the OS of patients treated with chemotherapy versus patients treated with ICI-chemo in the tumor-informed ctDNA-/ tumor-naïve ctDNA- group

**G.** Kaplan–Meier curve comparing the OS of patients treated with chemotherapy versus patients treated with ICI-chemo in the tumor-informed ctDNA+/ tumor-naïve ctDNA- group

**H.** Kaplan–Meier curve comparing the OS of patients treated with chemotherapy versus patients treated with ICI-chemo in the tumor-informed ctDNA+/ tumor-naïve ctDNA+ group

**I.** Kaplan–Meier curve comparing the OS of patients treated with ICI-chemo versus chemotherapy in patients with MSAF less than 1%, indicating a low concentration of ctDNA, stratified by ctDNA status. Hazard ratios (HRs) and corresponding 95% confidence intervals (CIs) were estimated using the Cox proportional hazards model. P values for survival differences were calculated using the two-sided log-rank test.


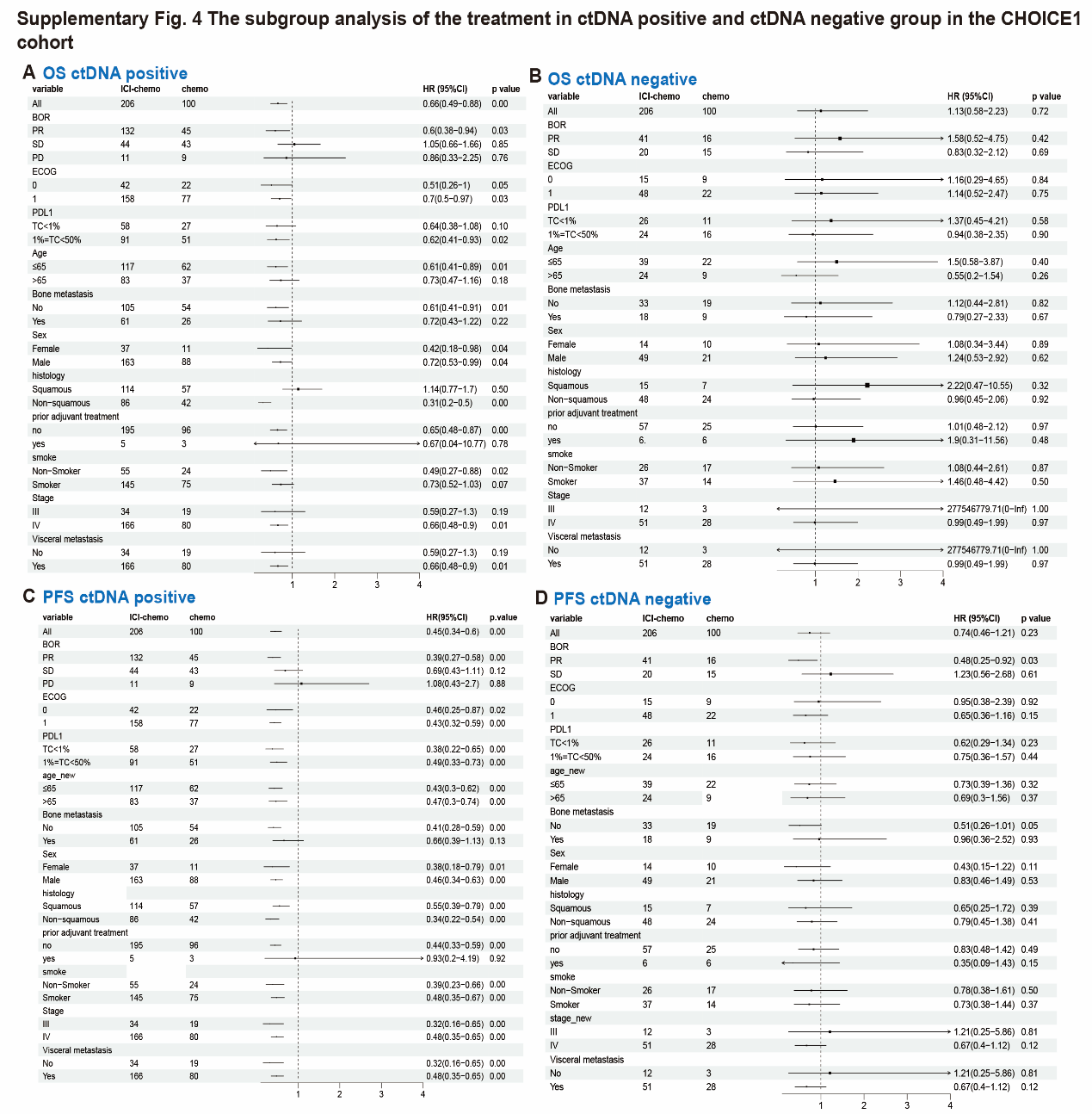
**Supplementary Fig. 4. The subgroup analysis of the therapy efficacy of ICI-chemo versus chemo in tumor-informed positive ctDNA or tumor-informed negative ctDNA group in the CHOICE-01 cohort**

**A-B.** Subgroup analysis of Overall Survival (OS) between the ICI-chemo and chemotherapy groups in tumor-informed ctDNA positive patients or tumor-informed ctDNA negative patients.

**C-D.** Subgroup analysis of Progression-Free Survival (PFS) between the ICI-chemo and chemotherapy groups in tumor-informed ctDNA positive patients or tumor-informed ctDNA negative patients. The hazard ratios, corresponding 95% confidence intervals, and statistical significance of the difference were computed using the Cox proportional hazards model.

**
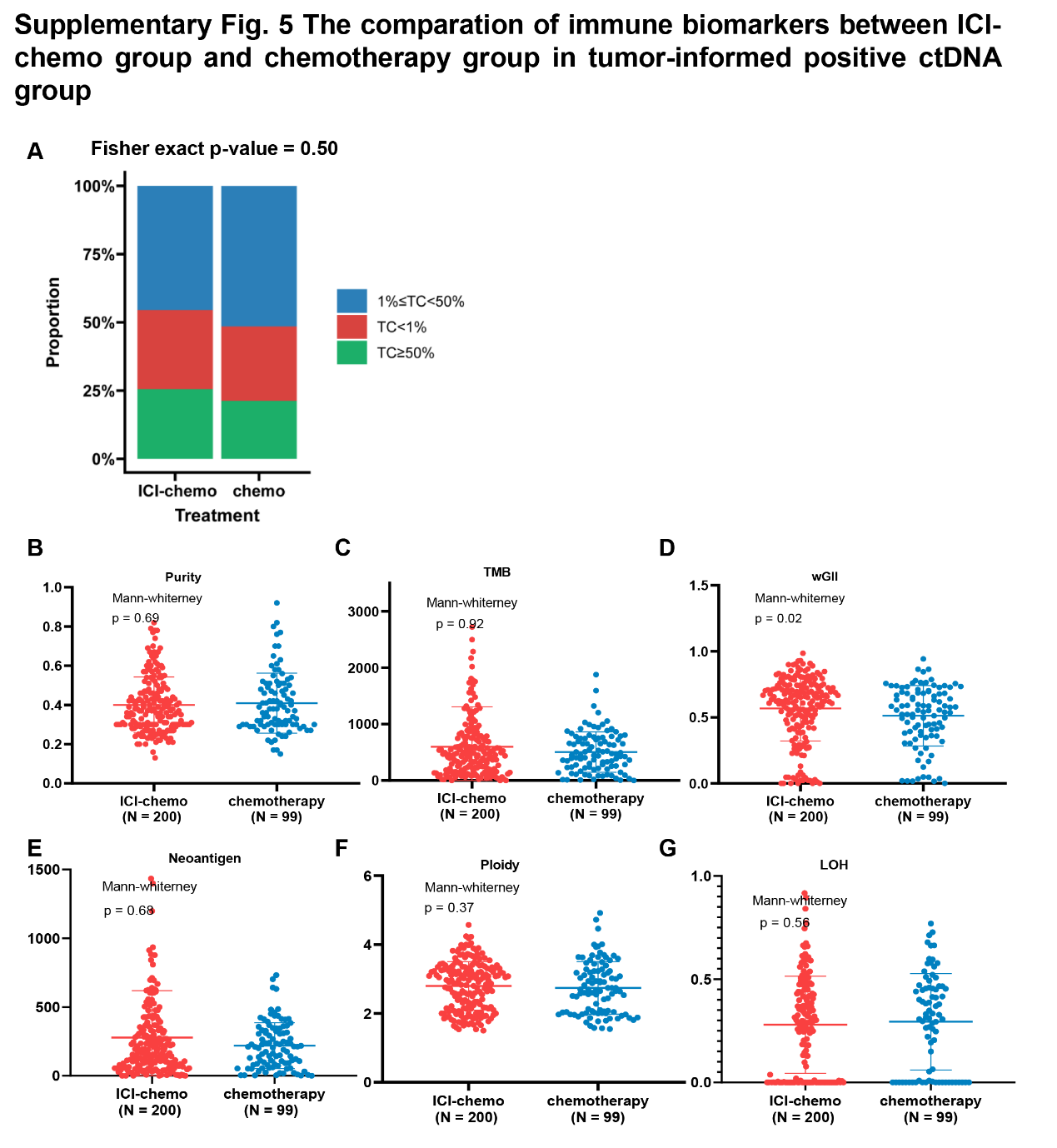
Supplementary Fig. 5. The immune biomarker between ICI-chemotherapy and chemotherapy group in the tumor-informed positive ctDNA group**

**A.** Comparison of patients with Programmed Cell Death 1 Ligand 1 (PD-L1) expression (Tumor Cell (TC) <1%, TC≥50%, 1%≤TC<50%) between the ICI-chemo group and the ICI group. P values were compared using Fisher's exact test.

**B-G.** Violin diagrams depicting the differences in tumor mutational burden (TMB), whole genome instability (wGII), tumor neoantigen burden (TNB), purity, ploidy, and loss of heterozygosity (LOH) between the ICI-chemo and chemotherapy groups. P values were compared using the Mann-Whitney test.


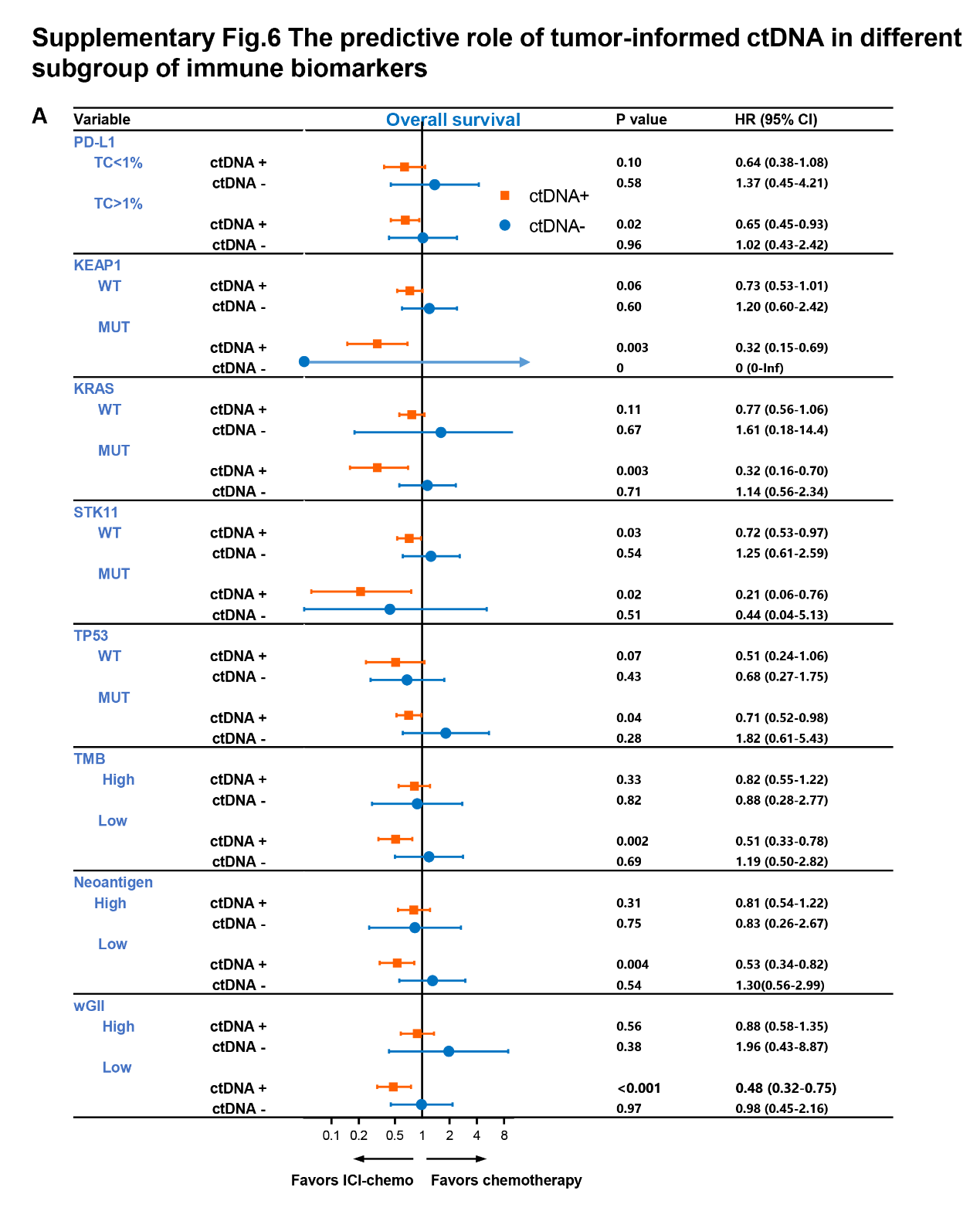


**Supplementary Fig. 6. The predictive role of tumor-informed ctDNA in the context of immune biomarkers**

**A.** Forest plot depicting the predictive role of tumor-informed ctDNA (ctDNA positive: ctDNA+; ctDNA negative: ctDNA-) for predicting Overall Survival (OS) of ICI-chemo versus chemotherapy stratified by the different subgroups of immune biomarkers The hazard ratios, corresponding 95% confidence intervals, and statistical significance of the difference were computed using the Cox proportional hazards model.

**
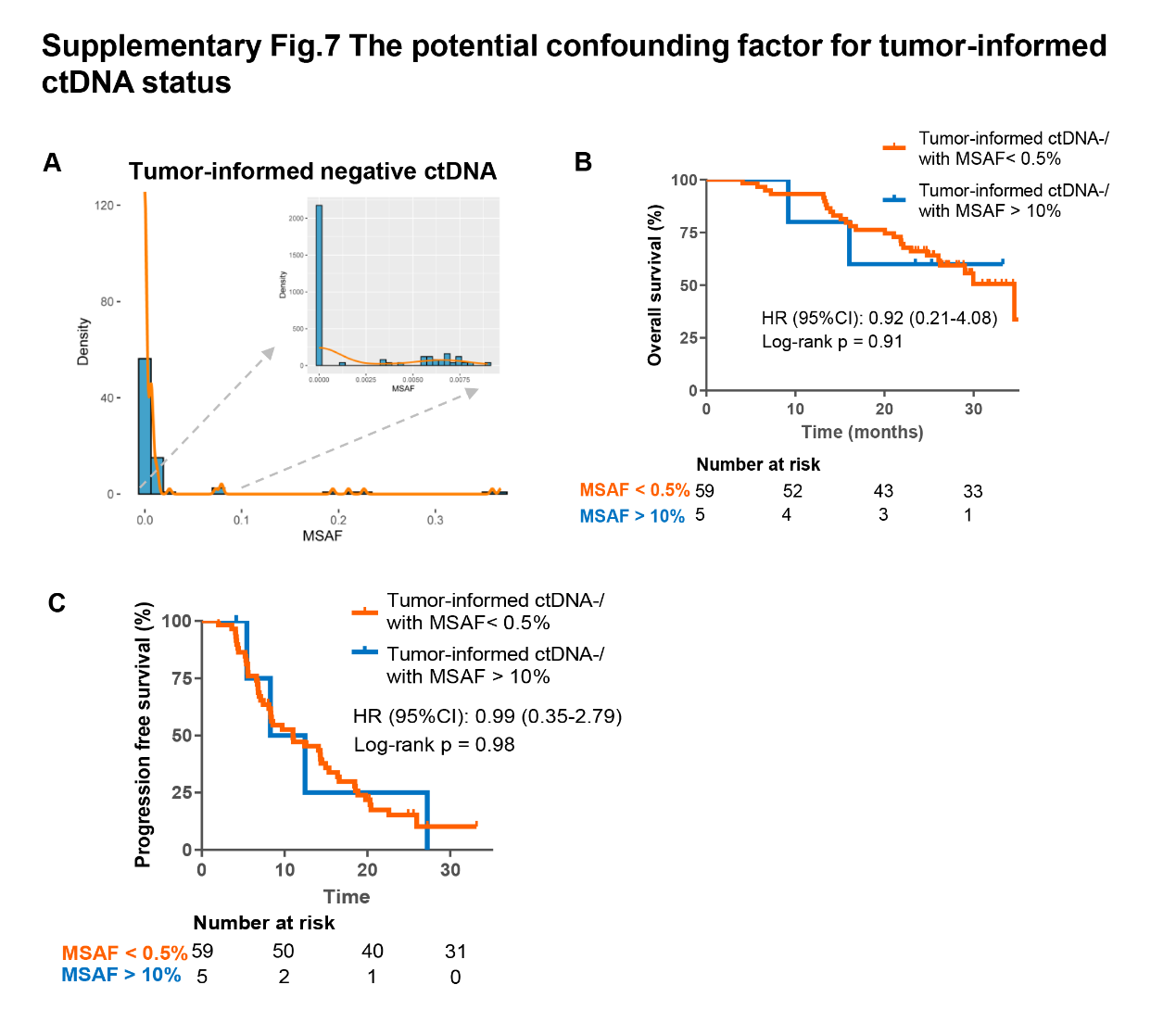
**

**Supplementary Fig. 7. The potential confounding factor for tumor-informed ctDNA status**

**A.** Density histogram illustrating the distribution of maximum somatic allele frequency (MSAF) in the tumor-informed negative circulating tumor DNA (ctDNA) group.

**B.** Kaplan–Meier estimates of Overall Survival (OS) comparing patients with MSAF > 20% and MSAF < 0.5% in the tumor-informed ctDNA negative group.

**C.** Kaplan–Meier estimates of Progression-Free Survival (PFS) comparing patients with MSAF > 20% and MSAF < 0.5% in in the tumor-informed ctDNA negative group. Hazard ratios (HRs) and corresponding 95% confidence intervals (CIs) were estimated using the Cox proportional hazards model. P values for survival differences were calculated using the two-sided log-rank test.

**
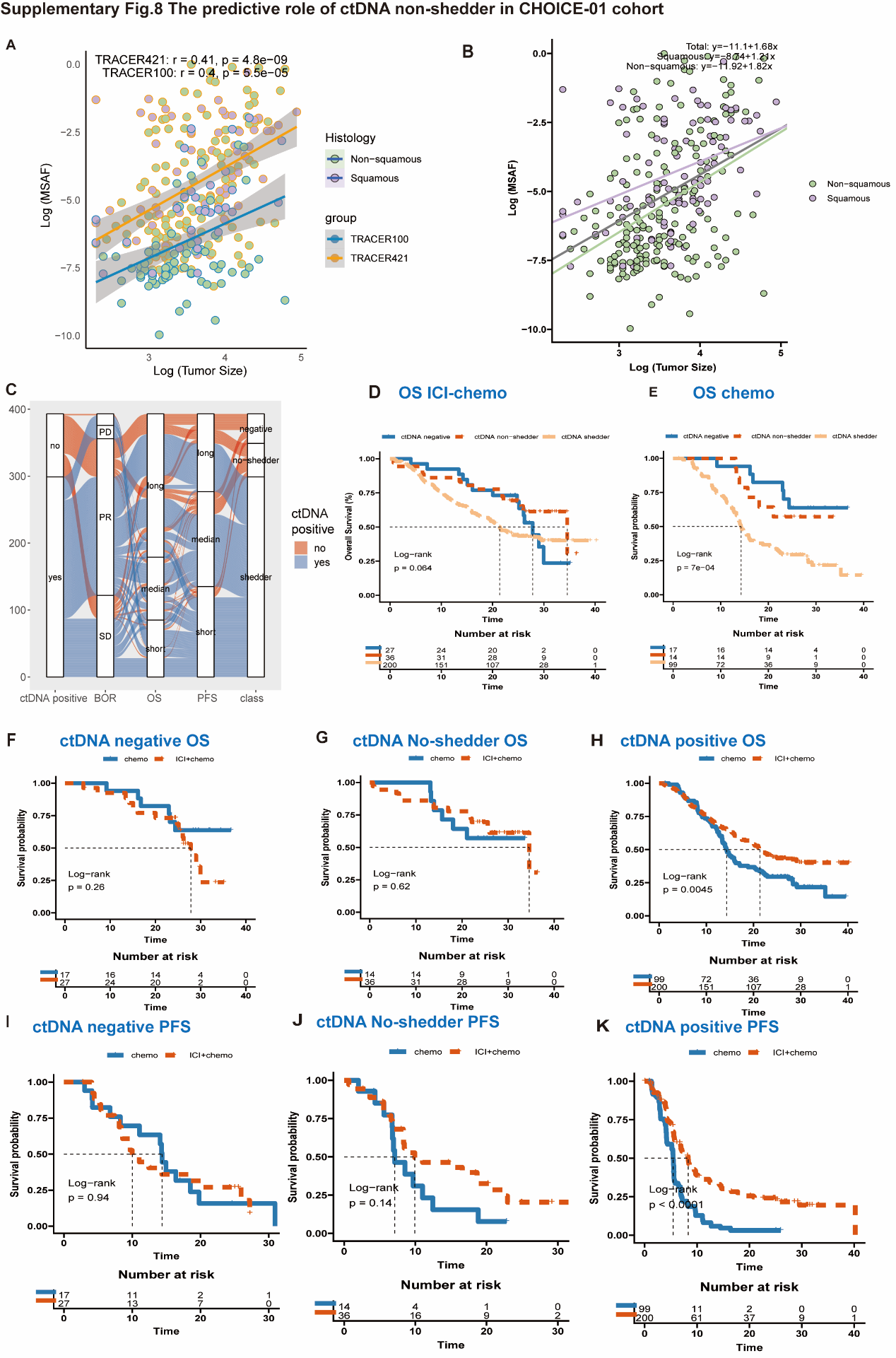
Supplementary Fig. 8. The predictive role of ctDNA non-shedder in CHOICE-01 cohort**

**A.** The log10-transformed tumor size versus log10-transformed maximum somatic allele frequency (MSAF) > 0.5% plot for individual case in CHOICE-01 study, colored by adenocarcinoma status and by squamous or other histology in TRACER100 study and TRACER421 studies. Fitted line represents a linear model line categorized by tumor histology. P values indicate adjusted linear model P values. Gray shaded area represents the 95% confidence interval for estimated MSAF based on tumor size.

**B.** Linear graph plotting the fitted linear function based on the log10-transformed tumor size versus log10-transformed MSAF.

**C.** Snaky plot depicting the association between tumor-informed ctDNA status and radiographic response BOR, Progression-Free Survival (PFS) (defined as long [PFS > 12 months], median [6 months < PFS ≤ 12 month], and short [PFS ≤ 6 months]), Overall Survival (OS) (defined as long [OS > 30 months], median [15 months <PFS ≤ 30 months], and short [OS ≤ 15 months]) and class (defined as ctDNA shedder, no-shedder, or ctDNA negative)

**D-E.** Kaplan–Meier estimates of OS comparing patients who were ctDNA shedders, ctDNA no-shedders and ctDNA negative in the ICI-chemo or chemo group.

**F-H.** Kaplan–Meier estimates of OS comparing patients who were treated with ICI-chemo and those who were treated with chemo in ctDNA shedders, ctDNA no-shedders and ctDNA negative population.

**I-K.** Kaplan–Meier estimates of PFS comparing patients who were treated with ICI-chemo and those who were treated with chemo in ctDNA shedders, ctDNA no-shedders and ctDNA negative population.


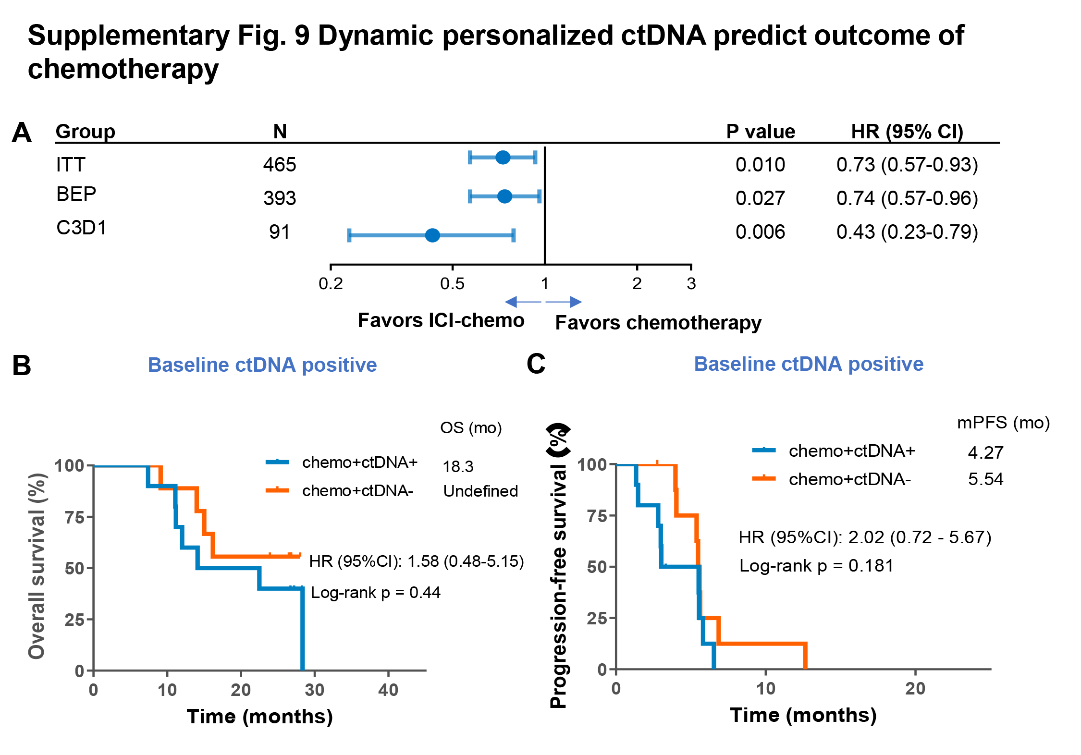
**Supplementary Fig. 9. Dynamic personalized ctDNA status predict superior outcome of ICI-chemotherapy**

**A.** Forest plots showing Hazard Ratios (HRs) and 95% Confidence Intervals (CI) for ICI-chemo versus chemotherapy in intent-to-Treat (ITT) population and the biomarker evaluable population (BEP) and C3D1 patient groups.

**B.** Kaplan–Meier estimates of Overall Survival (OS) comparing different ctDNA dynamic change groups among patients treated with chemotherapy in ctDNA positive group

**C.** Kaplan–Meier estimates of Progression-Free Survival (PFS) comparing different ctDNA dynamic change groups among patients treated with chemotherapy in ctDNA positive group

**
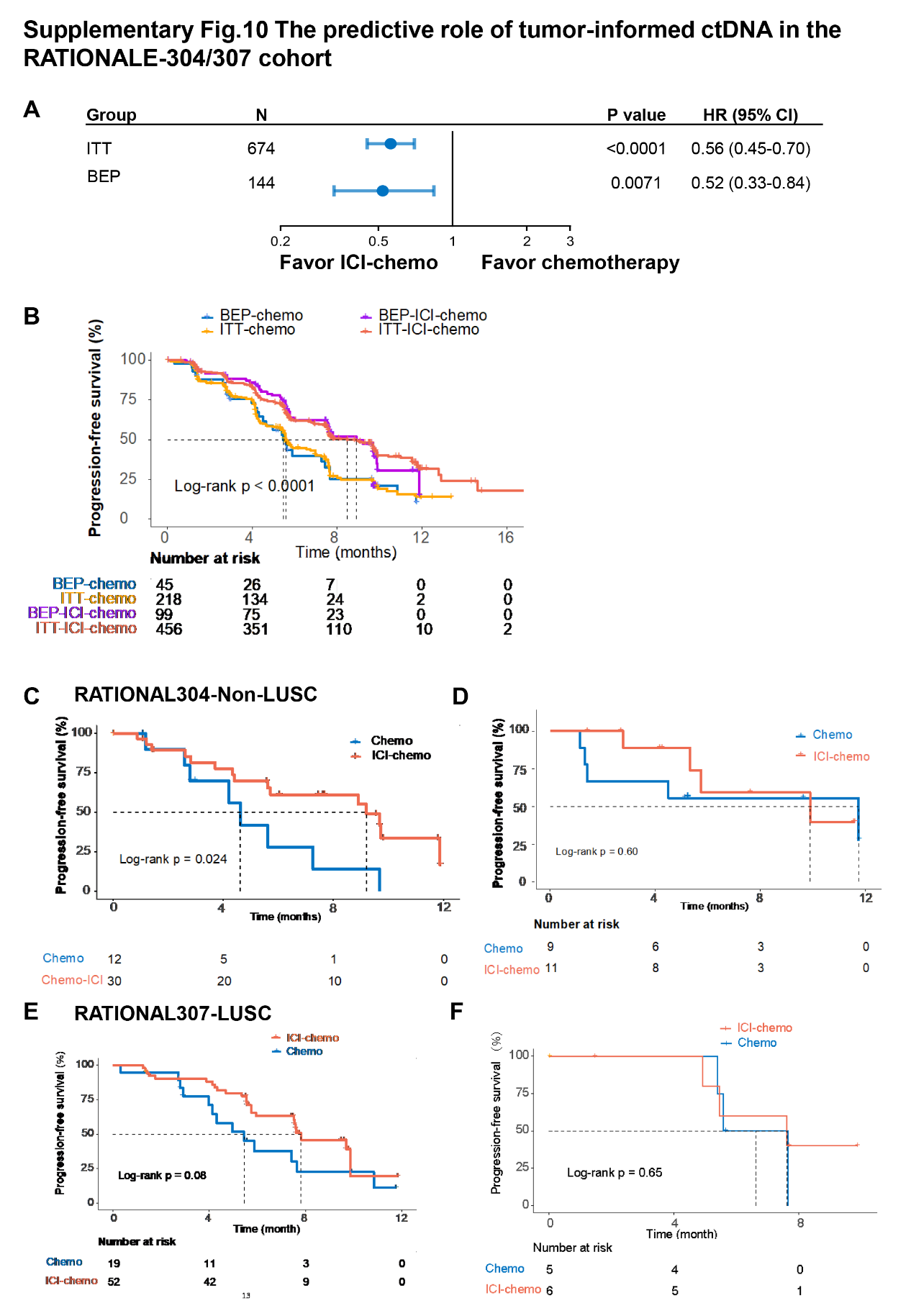
Supplementary Fig. 10. The predictive role of tumor informed ctDNA in the RATIONALE-304/307 cohort**

**A.** Forest plots displaying Hazard Ratios (HRs) and 95% Confidence Intervals (CIs) comparing the ICI-chemo versus chemotherapy in the patients of intent-to-Treat (ITT) population and the biomarker evaluable population (BEP) in RATIONALE 304/307 study.

**B.** Kaplan–Meier estimates of Progression-Free Survival (PFS) among patients treated with chemo-ICI and chemotherapy, stratified by tumor-informed ctDNA positivity or negativity.

**C.** Kaplan–Meier estimates of PFS among patients treated with chemo-ICI versus chemotherapy alone in patients with ctDNA-negative or ctDNA-positive status in non-lung squamous cell carcinoma (non-LUSC). Upper panel, Kaplan-Meier curves depicting the treatment comparison; lower, forest plot depicting the relative efficacy of chemo-ICI versus chemotherapy alone.

**E-F.** Kaplan–Meier estimates of PFS among patients treated with chemo-ICI versus chemotherapy alone in patients with ctDNA negative or ctDNA positive status in lung squamous cell carcinoma (LUSC). Upper panel, Kaplan-Meier curves depicting the treatment comparison; lower panel, forest plot depicting the relative efficacy of chemo-ICI versus chemotherapy alone.
